# Anlotinib as a third-line or further treatment for recurrent or metastatic nasopharyngeal carcinoma: a single-arm, phase 2 clinical trial

**DOI:** 10.1186/s12916-023-03140-x

**Published:** 2023-11-07

**Authors:** Yu Fang, Ning Su, Qihua Zou, Yi Cao, Yi Xia, Linquan Tang, Xiaopeng Tian, Panpan Liu, Qingqing Cai

**Affiliations:** 1 Department of Medical Oncology, Sun Yat-Sen University Cancer Center, Guangzhou, 510060 People’s Republic of China; 2 State Key Laboratory of Oncology in South China, Guangdong Key Laboratory of Nasopharyngeal Carcinoma Diagnosis and Therapy, Sun Yat-Sen University Cancer Center, Guangzhou, 510060 People’s Republic of China; 3 Department of Oncology, Sun Yat-Sen Memorial Hospital, Sun Yat-Sen University, Guangzhou, 510120 People’s Republic of China; 4 Department of Oncology, Guangzhou Chest Hospital, Guangzhou, 510095 People’s Republic of China; 5 Department of Nasopharyngeal Carcinoma, Sun Yat-Sen University Cancer Center, Guangzhou, 510060 People’s Republic of China

**Keywords:** Anlotinib, Nasopharyngeal carcinoma, Recurrent, Metastatic, Antiangiogenic therapy

## Abstract

**Background:**

Treatment options beyond the first-line setting for recurrent or metastatic nasopharyngeal carcinoma (RM-NPC) are limited. The role of the multitarget tyrosine kinase inhibitor anlotinib in RM-NPC is unclear.

**Methods:**

In this prospective, single-arm, phase 2 trial, patients with histologically confirmed RM-NPC and failure of at least two lines of prior systemic treatments were eligible. Anlotinib was given at 12 mg once daily on days 1–14 every 3 weeks until disease progression or intolerable toxicities. The primary end point was disease control rate, defined as the percentage of patients achieving complete response, partial response, or stable disease by RECIST criteria.

**Results:**

From April 2019 to March 2021, 39 patients were enrolled and received a median of 4 cycles (range, 0.5–20) of anlotinib treatment. Partial response and stable disease were observed in 8 and 20 patients, respectively. The disease control rate was 71.8%, and objective response rate was 20.5%. With a median follow-up of 17.2 months, the median progression-free survival was 5.7 months. The 12-month overall survival was 58.3%, and the median overall survival was not reached. The most frequent grade 3/4 treatment-related adverse events were hand-foot syndrome (23.7%), oral mucositis (21.0%), hypertension (7.9%), and triglyceride elevation (7.9%). Hemorrhage, all grade 1 or 2, occurred in 34.2% of the patients.

**Conclusions:**

Anlotinib monotherapy exhibited promising anti-tumor activities and disease control for heavily pretreated RM-NPC patients with a tolerable toxicity profile.

**Trial registration:**

ClinicalTrials.gov: NCT03906058.

**Supplementary Information:**

The online version contains supplementary material available at 10.1186/s12916-023-03140-x.

## Background

Nasopharyngeal carcinoma (NPC) ranks 23^rd^ in cancer prevalence globally [1], with new cases arising predominantly in east and southeast Asia. Approximately 10 to 15% of NPC patients have primary metastasis at the time of diagnosis, and 30% will develop local recurrence or distant metastases despite the standard first-line therapeutic regimens [2, 3]. Although cisplatin/gemcitabine (GP)-based regimens have been recommended as the first-line systemic treatment for recurrent or metastatic (RM)-NPC patients [4], patients who are refractory or progress after first-line treatment have few treatment options. Currently, there is no standard therapy in the second or subsequent line setting for RM-NPC. Therefore, a need exists for novel agents or therapeutic modalities for this refractory disease that carries a grave prognosis.

Antiangiogenic therapy hold promise for RM-NPC. NPC is amenable to antiangiogenic therapy as vascular endothelial growth factor (VEGF) is highly expressed in NPC and correlates with survival [5]. However, the addition of anti-VEGF antibody bevacizumab to paclitaxel plus carboplatin as first-line treatment failed to extend the progression-free survival (PFS) and overall survival (OS) of RM-NPC patients [6]. Other antiangiogenic multikinase inhibitors (MKIs) achieved an objective response rate (ORR) between 6.1 and 20% as monotherapy for pretreated RM-NPC [7–9]. Safety concerns such as hemorrhage have also hampered the use of antiangiogenic therapies for RM-NPC [10–12]. These observations suggest that the exploration of novel antiangiogenic MKIs for RM-NPC is necessary.

Anlotinib is a tyrosine kinase inhibitor that targets vascular endothelial growth factor receptor (VEGFR), fibroblast growth factor receptor (FGFR), platelet-derived growth factor receptors (PDGFR), and c-kit [13, 14]. It has been approved as 3rd line treatment for locally advanced or metastatic non-small cell lung cancer and 2nd line treatment for advanced soft-tissue sarcoma in China [15, 16]. Anlotinib has a low half-maximal inhibitory concentration (IC_50_) (0.2 nM) and has potent inhibitory activities on FGFR [13, 17]. It has been investigated in a variety of advanced tumors [18–21] but has not been examined in RM-NPC. This trial investigated the efficacy and safety of anlotinib in pretreated RM-NPC patients. An exploratory analysis was also undertaken to identify prognostic factors for antiangiogenic therapy for RM-NPC.

## Methods

### Patients

This single arm, phase II trial (ClinicalTrials.gov: NCT03906058) enrolled patients aged 18–70 years with pathologically confirmed locally recurrent or metastatic NPC and disease progression after at least two lines of prior systemic treatments. Patients had to have adequate organ function, an Eastern Cooperative Oncology Group (ECOG) performance status score of 0 or 1, and at least one measurable lesion per RECIST v1.1. Patients were excluded if they had received prior treatment with bevacizumab or VEGFR inhibitors or had received re-irradiation at nasopharyngeal lesions. Detailed eligibility criteria are described in the study protocol.

The trial was conducted according to the provisions of the Declaration of Helsinki and the International Conference on Harmonisation guidelines for Good Clinical Practice and approved by the institutional review board of Sun Yat-Sen University Cancer Center. All patients provided written informed consent before enrollment. The study protocol adhered to the SPIRIT statement and the reporting of the study adhered to the CONSORT statement.

### Treatment and assessments

Patients received 12 mg of anlotinib (Chia-tai Tianqing Pharmaceutical Co., Ltd.), orally once daily (2 weeks on and 1 week off), until disease progression or intolerable toxicities. Dose modification was allowed, with two levels of reduction (from 12 mg/day to 10 mg/day and 8 mg/day), for anlotinib-related toxicities according to protocol-specified criteria (generally for grade 3/4 toxicities). If more than 2 levels of dose modifications were required, treatment was terminated.

Responses were assessed by CT and MRI per RECIST v1.1 every two cycles during the anlotinib treatment. Adverse events (AEs) were graded according to the National Cancer Institute Common Terminology Criteria for Adverse Events (CTCAE) v5.0.

Plasma EBV DNA copy number was determined at baseline and every 3 weeks until disease progression by quantitative reverse transcription polymerase chain reaction with probes against EBV genes.

### Study end points

The primary end points were disease control rate (DCR), which was the proportion of patients achieving complete response (CR), partial response (PR), and stable disease (SD). The secondary end points included ORR, which was the proportion of patients achieving CR or PR; PFS, calculated from the date of study entry to progressive disease (PD) or death, whichever occurred earlier; OS, calculated from the date of study entry to death of all cause; and duration of response (DOR), which was the duration from initial CR or PR to PD or recurrence.

### Statistical analysis

A single-stage phase II design with a type I error of 5% and power of 80% was used to calculate the sample size. We considered the anlotinib treatment to be ineffective if DCR was ≤ 20% based on the result from the phase II study of gefitinib treatment for RM-NPC [22]. It has been reported that pazopanib treatment achieved a DCR of 54.5% (95% CI, 38.0–70.2) in RM-NPC patients (7). However, limited clinical data were available for anlotinib during the study's design phase. Considering the potential challenges of anlotinib tolerability in RM-NPC patients, which could subsequently impact treatment efficacy, we prudently selected a relatively conservative expected DCR of 40%. An estimated sample size of 35 patients was required. If at least 11 cases of CR or PR or SD were observed, the drug would be deemed effective. Assuming a dropout rate of 10%, the maximum estimated sample size was 39 patients.

All patients who received at least one dose of anlotinib were included in the efficacy and safety analysis set (full analysis set). No missing data was imputed. The 95% CIs of ORR and DCR were calculated using the Clopper-Pearson method. PFS and OS were calculated using the Kaplan–Meier method. Safety analysis mainly used descriptive statistics. All statistical analyses were performed using the SPSS 22.0 or GraphPad Prism software.

## Results

### Patient characteristics and treatment

**Fig. 1:**
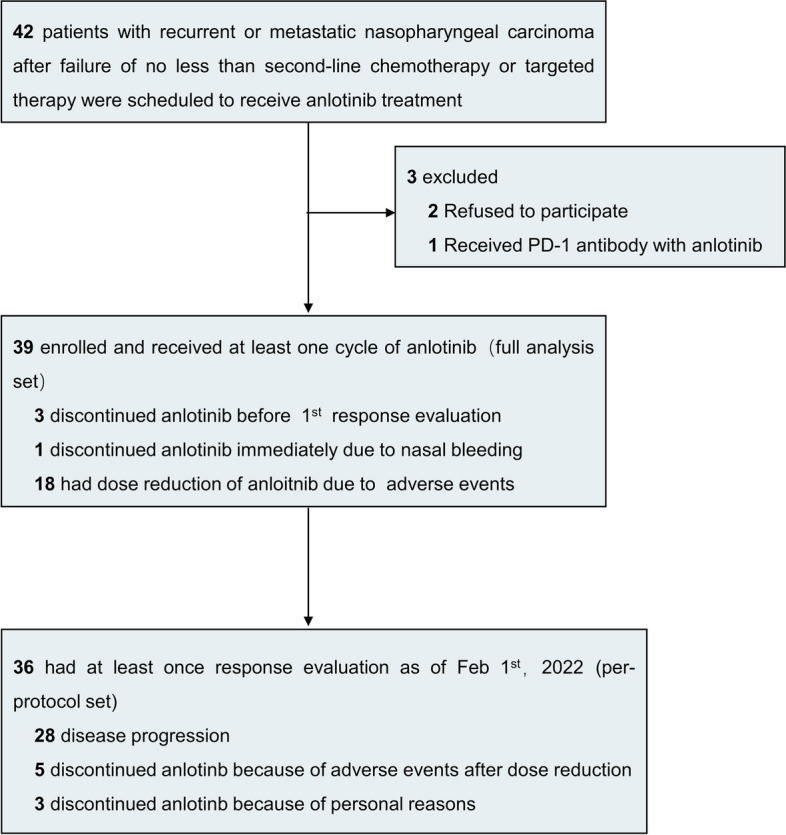
The study flowchart

Between March 2019 and March 2021, 42 RM-NPC patients were screened and 39 were eligible (Fig. 1), including 33 males (84.6%) and 6 females (15.4%) (Table 1). Their median age was 48 (range, 20–64) years. Twelve patients (30.8%) had locally recurrent disease at nasopharynx and/or cervical lymph nodes. Twenty-four patients (61.5%) had liver metastasis and 18 (46.2%) had lung metastasis. Thirty-five patients (89.7%) had received primary tumor loco-regional radiotherapy. Ten patients (25.6%) had received at least three prior lines of systemic therapy. Nineteen patients (48.7%) had received anti-PD-1 immunotherapy and 15 (38.5%) had received anti-epidermal growth factor receptor (EGFR) antibody. All 39 patients received at least one dose of anlotinib. By the data cutoff (April 30, 2022), they had received a median of 4 (range, 0.5–20) cycles of anlotinib.

**Table 1:** Patient demographic and baseline characteristics

Characteristic	*N* = 39
Age (years), median (range)	48 (20–64)
Sex	
Male	33 (84.6)
Female	6 (15.4)
ECOG performance status	
0	19 (48.7)
1	20 (51.3)
Histology	
Non-keratinizing	32 (82.1)
Undifferentiated (type III)	0
Differentiated (type II)	0
Keratinizing squamous cell carcinoma	7 (17.9)
Unclassified	
Disease stage	
Nasopharynx recurrent disease only	2 (5.1)
Cervical LN recurrent disease only	2 (5.1)
Nasopharynx and cervical LN recurrent disease	1 (3.6)
Nasopharynx recurrent and metastatic disease	1 (3.6)
Cervical LN recurrent and metastatic disease	6 (15.4)
Metastatic disease only	27 (69.2)
Metastasis type	
Oligometastasis	20 (51.3)
Polymetastases	19 (48.7)
Metastatic site	
Liver	24 (61.5)
Lung	18 (46.2)
Bone	17 (43.6)
Lymph node	32 (82.1)
EBV DNA	
≥ 1000 IU/mL	16 (41.0)
< 1000 IU/mL	23 (59.0)
Baseline LDH	
≥ 1 × ULN	13 (33.3)
< 1× ULN	26 (66.7)
Prior lines of systemic treatment^a^	
2L	29 (74.4)
3L	7 (17.9)
4L +	3 (7.7)
Previous systemic therapy	
Cisplatin	36 (92.3)
Carboplatin	3 (7.7)
Gemcitabine	31 (79.5)
Fluorouracil	14 (35.9)
Docetaxel	24 (61.5)
Paclitaxel	17 (43.6)
Capecitabine	16 (41.0)
S-1	22 (56.4)
Anti-PD-1 immunotherapy	19 (48.7)
Anti-EGFR antibody	15 (38.5)
Previous radiotherapy	35 (89.7)

Data are expressed as *N* (%) unless otherwise specified

*Abbreviations*: *LDH* Lactate dehydrogenase, *ULN* Upper limit of normal, *EBV* Epstein-Barr virus, *PD-L1* Programmed cell death protein-1, *EGFR* Epidermal growth factor receptor

^a^Concurrent chemoradiotherapy was considered as a first line of systemic treatment if the disease progressed within 6 months after the end of treatment

### Anti-tumor activities

**Fig. 2:**
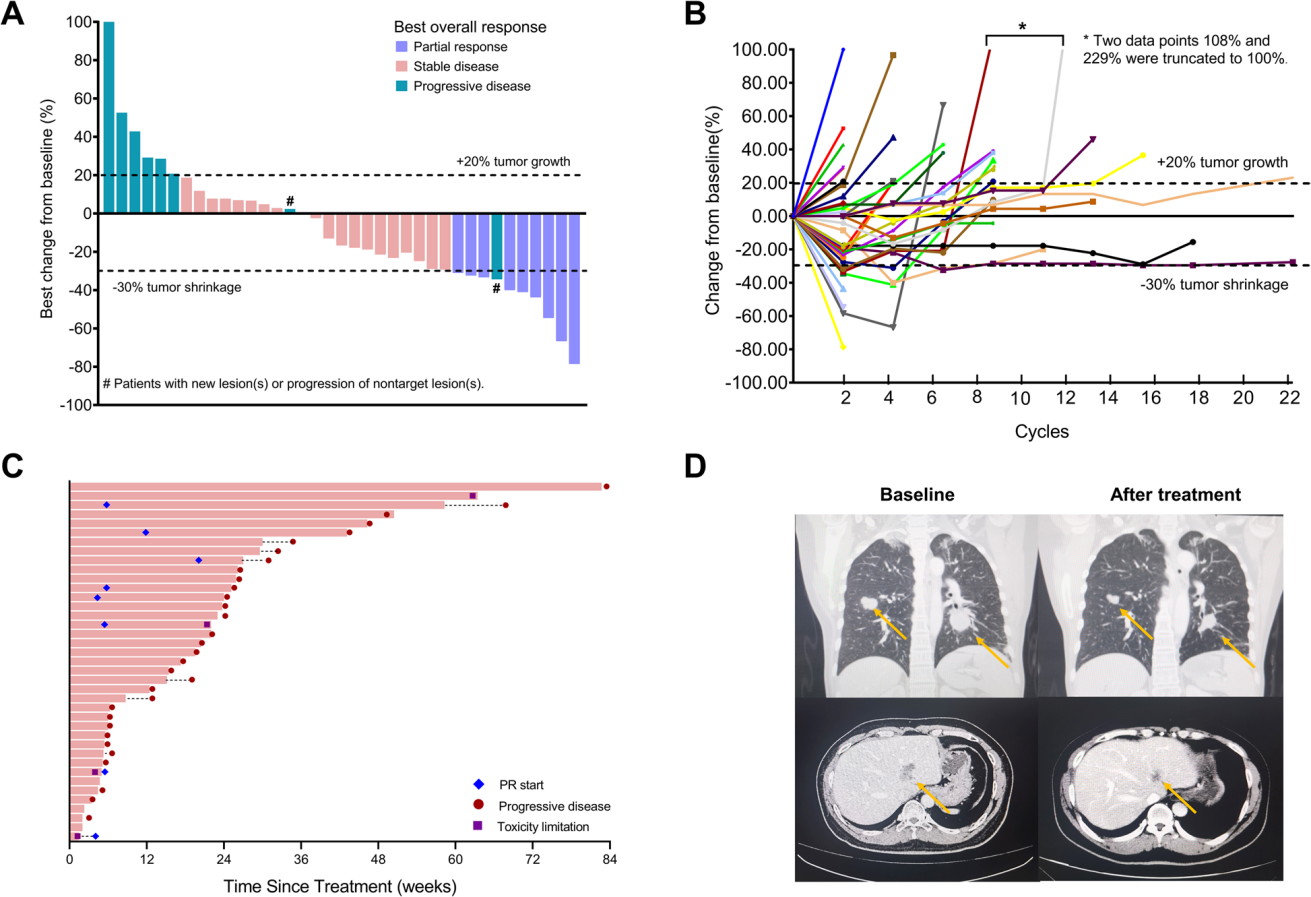
Tumor responses of 36 patients in the per-protocol set. **A** The waterfall plot shows the best percentage changes in target lesions from baseline according to Response Evaluation Criteria in Solid Tumours (RECIST), version 1.1. **B** The spider plot displays longitudinal changes from baseline in tumor size. The dotted lines at − 30% and 20% indicate partial response (PR) and progression per RECIST, respectively. **C** Treatment exposure and response duration. **D** Radiographic response by serial CT scans in pulmonary (upper panel) and hepatic (lower panel) metastases in nasopharyngeal carcinoma patients

No patient achieved CR and 8 patients attained PR. The ORR was 20.5% (95% CI, 9.3 to 36.5). Twenty patients had SD and the DCR was 71.8% (95% CI, 55.1 to 85.0) (Table 2). Twenty-one patients (58.3%) had a reduction in target lesion size (Fig. 2). The median time to treatment response was 1.4 months (95% CI, 0.9 to 3.0), and the median DOR was 4.0 months (95% CI, 1.0 to 7.1). Three patients discontinued anlotinib before the 1^st^ efficacy evaluation and were excluded from the per-protocol set (PPS). The ORR and DCR were similar in the PPS population (Table 2).

**Table 2:** Tumor response with anlotinib in RM-NPC according to RECIST v.1.1

Responses	FAS (*n* = 39)	PPS (*n* = 36)
Best overall response, no.; % (95% CI)		
CR	0; 0 (0 to 9.0)	0; 0 (0 to 9.7)
PR	8; 20.5 (9.3 to 36.5)	8; 22.2 (10.1 to 39.2)
SD	20; 51.3 (34.8 to 67.6)	20; 55.6 (38.1 to 72.1)
PD	8; 20.5 (9.3 to 36.5)	8; 22.2 (10.1 to 39.2)
NA	3; 7.7 (1.6 to 20.9)	0; 0 (0 to 9.7)
ORR, *n* (%)	8; 20.5 (9.3 to 36.5)	8; 22.2 (10.1 to 39.2)
DCR, *n* (%)	28; 71.8 (55.1 to 85.0)	28; 77.8 (60.8 to 89.9)

*CR* Complete response, *DCR* Disease control rate, *FAS* Full analysis set, *NA* Not assessable, *ORR* Objective response rate, *PD* Progressive disease, *PPS* Per-protocol set, *PR* Partial response, *SD* Stable disease

**Fig. 3:**
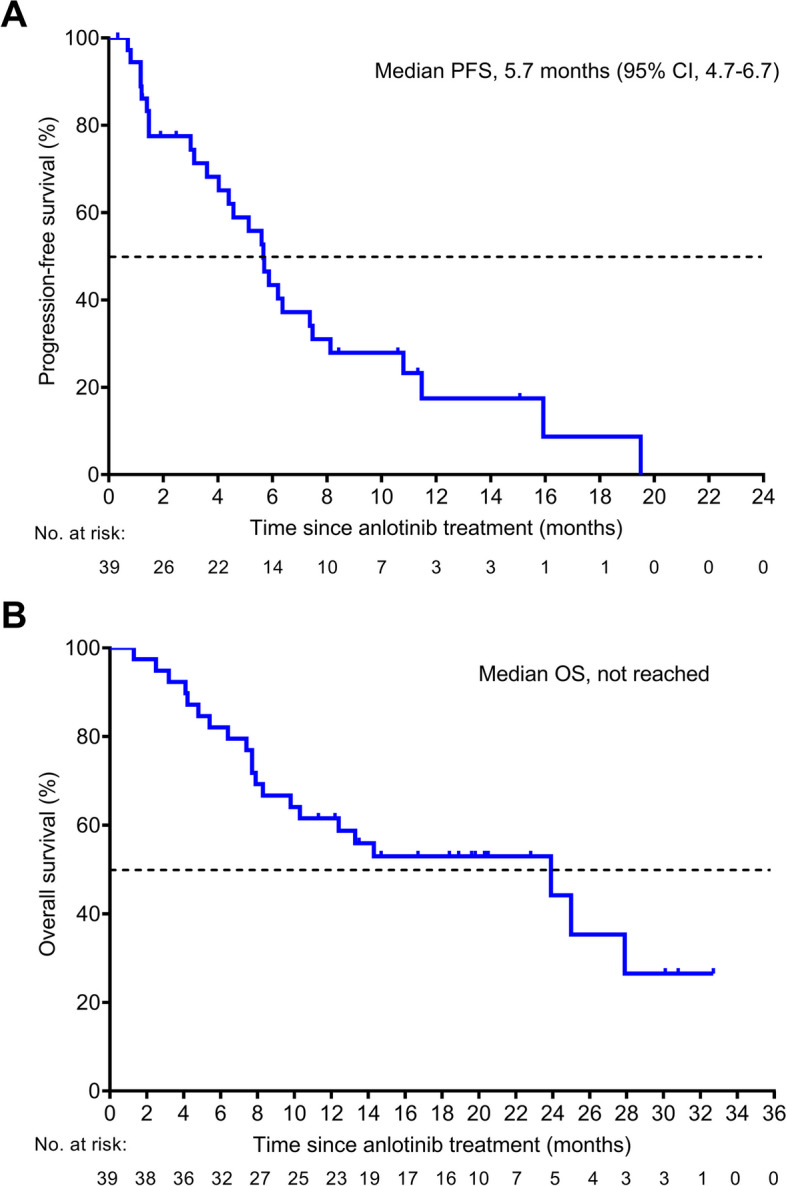
Survival outcomes in patients with RM-NPC treated with anlotinib. **A** Progression-free survival (PFS). **B** Overall survival (OS). Number of patients at risk at indicated time points are shown beneath the *x-*axis

The median duration of follow up was 17.2 (range, 1.3–32.7) months. Twenty-eight PFS events occurred, and the median PFS was 5.7 months (95% CI, 4.7 to 6.8) (Fig. 3A). The 6-month PFS rate was 36.1%. At the data cutoff, 21 deaths were reported. The 12-month OS was 58.3%, and the median OS was not reached (Fig. 3B).

### Safety

Thirty-eight patients were included in the safety set. All patients had treatment-related AEs (TRAEs). The most frequent TRAEs of all grades were hand-foot syndrome (HFS, 63.2%), hypothyroidism (60.5%), hypertension (55.3%), oral mucositis (47.4%), and cholesterol elevation (43.6%) (Table 3). The most frequent grade 3/4 TRAEs were hand-foot syndrome (HFS, 23.7%), oral mucositis (21.0%), hypertension (7.9%), and triglyceride elevation (7.9%). Hemorrhage occurred in 34.2% of the patients, and all were grade 1/2. Nineteen patients (50%) had dose reduction due to TRAEs. Additionally, 4 patients (10.5%) experienced treatment interruptions due to TRAEs. Four patients (10.5%) discontinued anlotinib due to TRAEs, including pharyngeal necrosis (*n* = 2), HFS (*n* = 1), and bleeding (*n* = 1). No treatment-related death was reported.

**Table 3:** Frequent treatment-related adverse events occurring in ≥ 5% of the study patients (*N* = 38)

Adverse events	All grades	Grade 3 or higher
TRAEs		
Serious TRAE	1 (2.6)	
Leading to anlotinib dose reduction	19 (50.0)	
Leading to anlotinib treatment interruption	4 (10.5)	
Leading to treatment termination	4 (10.5)	
Anlotinib-related AEs causing death	0	
Hand-foot syndrome^a^	24 (63.2)	9 (23.7)
Hypothyroidism	23 (60.5)	0
Hypertension	21 (55.3)	3 (7.9)
Oral mucositis^b^	18 (47.4)	8 (21.1)
Cholesterol elevation	17 (43.6)	0
Triglyceride elevation	15 (39.5)	3 (7.9)
Bleeding^c^	13 (34.2)	0
Proteinuria	13 (34.2)	0
Fatigue	10 (25.6)	0
GGT elevation	10 (26.3)	2 (5.3)
Anemia	9 (23.7)	2 (5.3)
Creatine elevation	8 (21.1)	2 (5.3)
Anorexia	7 (18.4)	0
AST elevation	5 (13.2)	1 (2.6)
ALT elevation	4 (10.5)	1 (2.6)
Nausea	3 (7.9)	0
Neutropenia	3 (7.9)	1 (2.6)
TBIL elevation	2 (5.3)	1 (2.6)
Pharynx necrosis	2 (5.3)	2 (5.3)
Rash	1 (2.6)	0

Data are expressed in *N* (%)

There were two cases of pharyngeal necrosis (1 grade 3; 1 asymptomatic and intervention not indicated)

*AE* Adverse event, *ALT* Alanine aminotransferase, *AST* Aspartate aminotransferase, *GGT* Gamma-glutamyl transpeptidase, *TBIL* Total bilirubin, *TRAE* Treatment-related adverse event

^a^For hand-foot syndrome, 7 patients with grade 3 TRAE lead to anlotinib dose reduction or treatment interruption

^b^For oral mucositis, 6 grade 3 events required anlotinib dose reduction

^c^Sites of grade 1–2 bleeding were the nasopharynx (*n* = 8), the oropharynx (*n* = 2), and the urinary tract (*n* = 3)

### Subgroup analysis

Patients who had received prior anti-PD-1 immunotherapy (*n* = 19, 48.7%) had a higher ORR (26.3% *vs.* 15.0%) and DCR (78.9% *vs.* 65.0%) than those had not received anti-PD-1 immunotherapy (*n* = 20, 51.3%). The duration of DCR was also longer in patients who had received prior anti-PD-1 immunotherapy versus those not (median duration of DCR, 24.3 weeks *vs*. 20.4 weeks) (Additional file 1: Fig S1). Five of the immunotherapy-exposed patients attained PR, and all of these patients finished anti-PD-1 immunotherapy less than 3 months before anlotinib treatment.

Patients with a baseline EBV DNA copy number < 1000 IU/mL (*n* = 22) had a higher DCR (61.1% *vs*. 38.8) and ORR (31.8% *vs*. 7.1%) than patients whose baseline EBV DNA copy number was ≥ 1000 IU/mL (*n* = 14) (Additional file 1: Fig S2A). The plasma EBV DNA copy number was dynamically monitored in 36 patients. Eighteen patients experienced a decline or no change from baseline, while 18 exhibited an increase in EBV DNA copy number after cycle 1. Patients with a decline or no change in EBV DNA copy number attained a higher DCR than those with an elevated EBV DNA copy number (94.4% *vs*. 61.1%) (Additional file 1: Fig S2B). Twenty-three patients developed HFS and had a higher DCR than those without HFS (91.3% *vs*. 53.8%) (Additional file 1: Fig S2A and C).

## Discussion

In this trial, RM-NPC patients who received anlotinib monotherapy as third-line or further therapy demonstrated effective disease control and acceptable toxicity profile. Subgroup analysis identified plasma EBV DNA levels and HFS as prognostic factors of treatment response. The findings support further clinical development of anlotinib for RM-NPC patients who have dismal survival outcomes and limited treatment options.

Several antiangiogenic MKIs had been studied in RM-NPC (Additional file 1: Table S1) [7–9, 11, 23, 24]. Early trials of sorafenib [23] and sunitinib [11] had shown modest efficacy in RM-NPC but significant risk of fatal hemorrhages. Moderate toxicity was observed during pazopanib [7] and axitinib [8] treatment, but the response rate was still poor, with an ORR of 3.7% to 6.1%. Recent study has reported higher efficacy with apatinib [24] and lucitanib [9] treatment, with an ORR of 20% to 36.4% and a DCR of 54.5% to 90%. However, patients enrolled in the trials mentioned above were resistant to traditional chemotherapy, and most of them had not been exposed to targeted therapy or immunotherapy. In this trial, nearly half (48.7%) of the patients had received anti-PD-1 immunotherapy, and over one third had received anti-EGFR antibody. Anlotinib monotherapy demonstrated an ORR of 20.5%, a DCR of 71.8%, and a median PFS of 5.7 months, which was comparable with the efficacy results of apatinib and lucitanib. These results suggested anlotinib as a promising agent for heavily pretreated NPC patients with both high clinical benefit rate and durable disease control in the era of targeted therapy and immunotherapy. Such an efficacy may be attributed to the high affinity and selectivity of anlotinib for targeted kinases [13, 14].

In this study, patients with prior anti-PD-1 immunotherapy tended to have higher therapeutic efficacy to anlotinib treatment, especially for patients who finished anti-PD-1 immunotherapy less than 3 months before anlotinib treatment. This may be because the anti-tumor immunity remains activated in the short term after the end of immunotherapy, while antiangiogenic drugs could further increase the infiltration of immune effector cells in the tumor microenvironment by normalizing immature blood vessels [25]. The synergistic anti-tumor effect of antiangiogenic therapy and immunotherapy has been indicated by preclinical studies [25] and validated by clinical trials in renal carcinoma [26–28], endometrial carcinoma [29], and hepatocellular carcinoma [30]. Recent phase II study [31] has demonstrated the promising therapeutic efficacy and safety of camrelizumab plus apatinib in patients with recurrent or metastatic NPC who failed first-line therapy, with an ORR of 65.5% and a DCR of 86.2%. The phase II TORAL trial (ClinicalTrials.gov: NCT04996758) evaluating the efficacy and safety of anlotinib in combination with the anti-PD-1 antibody toripalimab in RM-NPC is currently ongoing.

Grade 3/4 HFS and oral mucositis observed in this trial were more common than previously reported for anlotinib monotherapy in patients with advanced non-small cell lung cancer, soft-tissue sarcoma, and medullary thyroid carcinoma [18, 20, 21]. The higher incidence of HFS and oral mucositis in RM-NPC may be attributed to previous exposure to chemotherapeutic agents commonly used in NPC, including 5-fluorouracil, capecitabine, docetaxel, and other multikinase inhibitors, which can also cause reactions involving the hands, feet, and mucosa [32]. Previous radiotherapy may have also contributed to the higher incidence of oral mucositis. HFS is reversible in most cases but may impact quality of life. In this trial, HFS was associated with better outcomes in RM-NPC patients with anlotinib treatment. The prognostic value of HFS had also been reported in patients with other advanced malignancies treated with MKIs including anlotinib [33, 34]. One of the main concerns with antiangiogenic agents is the occurrences of bleeding. In this trial, grade 1/2 bleeding occurred in 34.2% of the patients; however, no patient had grade 3/4 bleeding. Considering the occurrence of fatal hemorrhage reported during sunitinib treatment in RM-NPC patients who had received high-dose radiation [11], bleeding should also be monitored closely during the treatment of anlotinib in patients with previous radiotherapy.

In this trial, high baseline EBV DNA copy number (≥ 1000 IU/mL) and increase from baseline in EBV DNA level were all associated with higher rate of disease progression during the anlotinib treatment. In the pilot study of single-agent apatinib treatment in RM-NPC patients, low baseline EBV DNA level was associated with long-term response, while change of EBV-DNA level after apatinib treatment was not correlated with the duration of response [35]. These findings suggested that antiangiogenic therapies may be more effective in preventing disease progression in patients with low disease burden. However, although the correlation between treatment response and EBV DNA changes has also been reported in bevacizumab treatment for RM-NPC patients [6], this observation may speak to the anti-tumor efficacy of these drugs on overall disease burden. Therefore, change of plasma EBV DNA level may not be a specific biomarker for the efficacy of antiangiogenic therapies but should still be monitored during antiangiogenic treatment in NPC patients.

The study has several limitations. First, this is a single-arm study in the absence of a control group. Second, the results of dynamic monitoring of EBV DNA copy numbers were not available in all enrolled patients. Thirdly, the results of our subgroup analysis may be limited by the small cohort size. Finally, only Han Chinese subjects were included in the trial. The efficacy and safety of anlotinib should be evaluated in NPC patients of diverse ethnicities across countries in the future.

## Conclusions

Anlotinib as a third- or later-line treatment is well tolerated and has demonstrated promising anti-tumor activity and disease control in heavily pretreated RM-NPC patients. The efficacy and safety of anlotinib in combination with immune checkpoint inhibitors for the treatment of RM-NPC patients should be further explored.

### Supplementary Information

**Additional file 1: Figure S1.** Treatment exposure and response duration of immunotherapy-exposed and immunotherapy-naïve patients. **Figure S2.** Subgroup Analysis. (A) Forest plot analysis of DCR by patient subgroups. (B) Best response and EBV DNA change. Changes in plasma EBV DNA copy number post cycle 1 from baseline (left panel) and treatment responses of patients (right panel). (C) Hand-foot syndrome in patients treated with anlotinib (left panel) and treatment responses of patients per occurrence of hand-foot syndrome (right panel). Patient responses are colored coded. EBV, Epstein-Barr virus; PD, progressive disease; PR, partial response; SD, stable disease. **Table S1.** Studies of antiangiogenic multikinase inhibitors in recurrent and metastatic nasopharyngeal carcinoma.

**Additional file 2.** Study protocol.

## Data Availability

The datasets used and analyzed during the current study are available on reasonable request from the corresponding author.

## Abbreviations

AEs: Adverse events
CR: Complete response
CTCAE: Common Terminology Criteria for Adverse Events
DOR: Duration of response
ECOG: Eastern Cooperative Oncology Group
EGFR: Epidermal growth factor receptor
FGFR: Fibroblast growth factor receptor
GP: Cisplatin plus gemcitabine
HFS: Hand-foot syndrome
IC50: Half-maximal inhibitory concentration
MKIs: Antiangiogenic multikinase inhibitors
NPC: Nasopharyngeal carcinoma
ORR: Objective response rate
OS: Overall survival
PD: Progressive disease
PDGFR: Platelet-derived growth factor receptor
PFS: Progression-free survival
PPS: Per-protocol set
PR: Partial response
RM-NPC: Recurrent or metastatic nasopharyngeal carcinoma
SD: Stable disease
TRAEs: Treatment-related AEs
VEGF: Vascular endothelial growth factor
VEGFR: Vascular endothelial growth factor receptor
