# Supplementary material for: Anlotinib as a third-line or further treatment for recurrent or metastatic nasopharyngeal carcinoma: a single-arm, phase 2 clinical trial
Source: BMC Med. 2023 Nov 7;21:423. doi: 10.1186/s12916-023-03140-x (PMC10631002; doi:10.1186/s12916-023-03140-x)
Supplement: Supplementary file 1 — Additional file 1: Figure S1. Treatment exposure and response duration of immunotherapy-exposed and immunotherapy-naïve patients. Figure S2. Subgroup Analysis. (A) Forest plot analysis of DCR by patient subgroups. (B) Best response and EBV DNA change. Changes in plasma EBV DNA copy number post cycle 1 from baseline (left panel) and treatment responses of patients (right panel). (C) Hand-foot syndrome in patients treated with anlotinib (left panel) and treatment responses of patients per occurrence of hand-foot syndrome (right panel). Patient responses are colored coded. EBV, Epstein-Barr virus; PD, progressive disease; PR, partial response; SD, stable disease. Table S1. Studies of antiangiogenic multikinase inhibitors in recurrent and metastatic nasopharyngeal carcinoma. [file 12916_2023_3140_MOESM1_ESM.pdf]

Anlotinib as a Third-Line or Further Treatment for Recurrent or Metastatic Nasopharyngeal Carcinoma: A Single-arm, Phase 2 Clinical Trial

Yu Fang, Ning Su, Qihua Zou, Yi Cao, Yi Xia, Linquan Tang, Xiaopeng Tian, Panpan Liu, Qingqing Cai

Table of Contents

Figure S1 1

Figure S2 2

Table S1 3

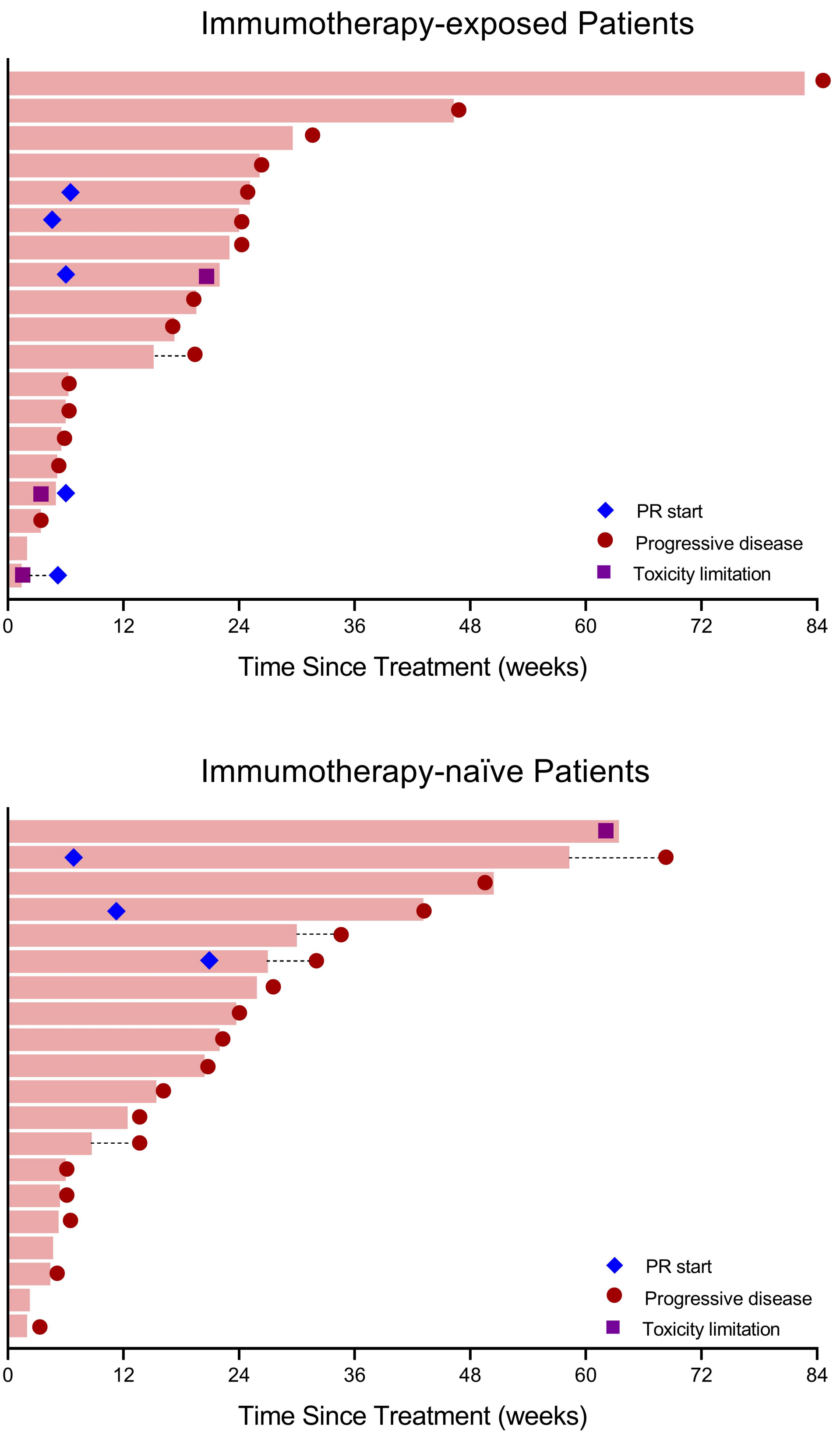

Figure S1. Treatment exposure and response duration of immunotherapy -exposed and immunotherapy-naïve patients.

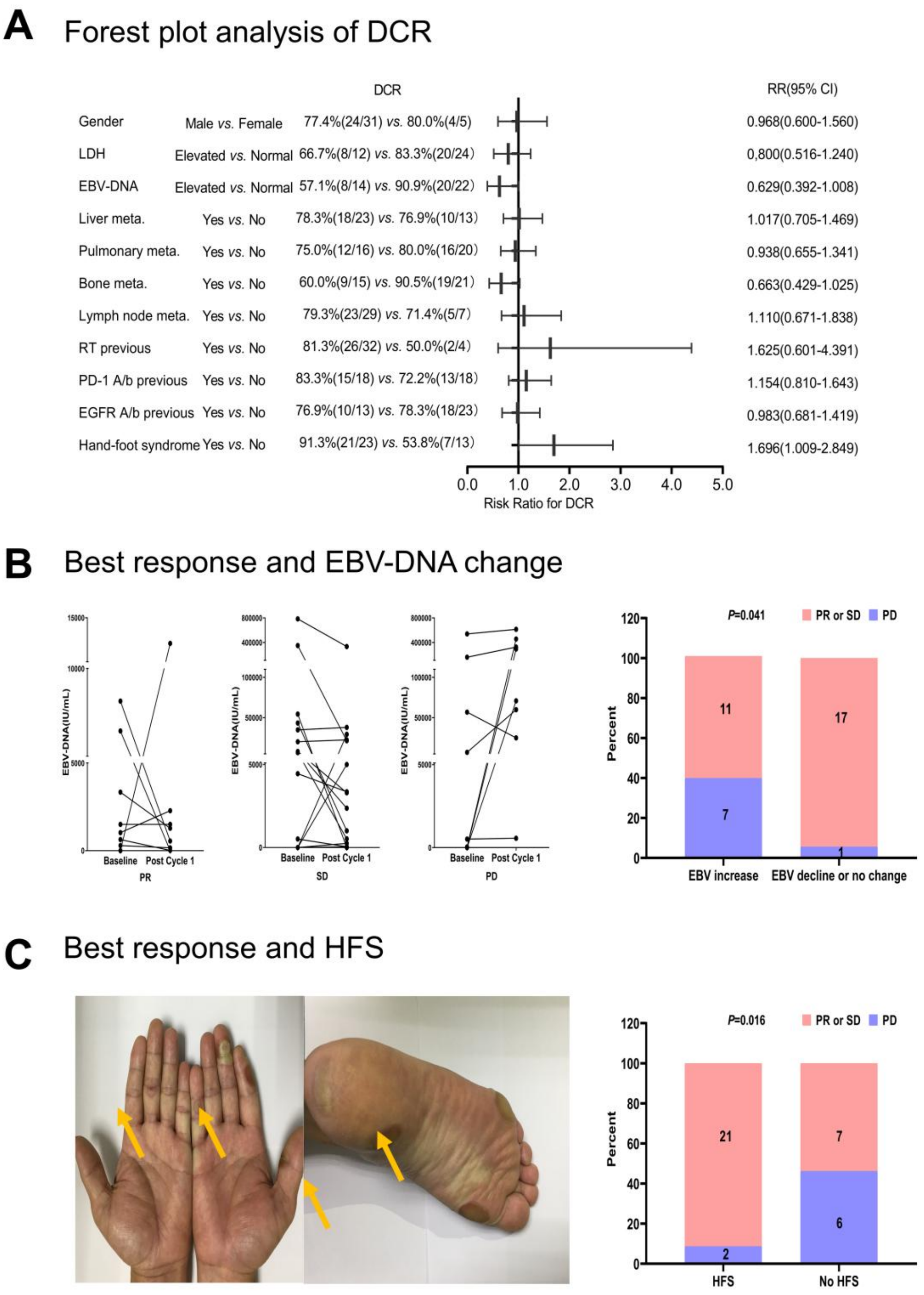

Figure S2. Subgroup Analysis. (A) Forest plot analysis of DCR by patient subgroups. (B) Best response and EBV-DNA change. Changes in plasma EBV DNA copy number post cycle 1 from baseline (left panel) and treatment responses of patients (right panel). (C) Hand-foot syndrome in patients treated with anlotinib (left panel) and treatment responses of patients per occurrence of hand-foot syndrome (right panel). Patient responses are colored coded. EBV, Epstein-Barr virus; PD, progressive disease; PR, partial response; SD, stable disease.

Table S1. Studies of antiangiogenic multikinase inhibitors in recurrent and metastatic nasopharyngeal narcinoma.

Agent	Type	Number	Efficacy	Toxicity
Sorafenib[23]	Prospective phase II	7 NPC + 20 SCCHN	ORR (3.7%), DCR (37.0%), mPFS (3.4 mos), mOS (4.2 mos).	Grade 3 lymphopenia (17%), fatigue (7%), hyponatremia (3%), leucopenia (3%), and lipase elevation (3%). One death for epistaxis.
Sunitinib[11]	Prospective phase II	13	ORR (10.0%), DCR (30.0%), mPFS (3.5 mos), mOS (10.5 mos).	Grade 3/4 hemorrhage (28.6%), leucopenia (28.6%), fatigue (21.4%), neutropenia (21.4%) and dysphagia (14.3%). Two death for grade 5 hemorrhage.
Pazopanib[7]	Prospective phase II	33	ORR (6.1%), DCR (54.5%) at 12 weeks, mPFS (4.4 mos), mOS (10.8 mos).	Grade 3/4 fatigue (15.2%), hand-foot syndrome (15.2%), anorexia (9.1%), diarrhea (6.1%), and vomiting (6.1%).
Axitinib[8]	Prospective phase II	40	ORR (3.7%), DCR (78.4%) at 3 months, mPFS (5.0 mos), mOS (10.4 mos).	Grade 3/4 hypertension (8%), diarrhea (5%), weight loss (5%), and pain (5%). All hemorrhage were grade 1 -2.
Apatinib[24]	Prospective phase II	33	ORR (36.4%), DCR (54.5%) at 5 months, mPFS (5.0 mos), mOS (16.0 mos).	Grade 3/4 hand-foot syndrome (15.2%), oral ulcer (15.2%), proteinuria (6.1%), leukopenia (6.1%), thrombocytopenia (6.1%) and hemorrhage (3.0%).
Lucitanib[9]	Prospective phase Ib	10 in the continuous arm,<br>10 in the continuous arm.	ORR (20%), DCR (90%), mPFS (3.73 mos) for the continuous arm; ORR (10%), DCR (60%), mPFS (3.68 mos) for the intermittent arm.	Grade 3/4 hypertension (30% vs 0%), proteinuria (20% vs 0%), increased AST (10% vs 0%), and decreased platelet count (10% vs 0%),

Anlotinib	Prospective phase II	39	ORR (20.5%), DCR (71.8%), mPFS (5.7 mos), mOS (NR), 12-month OS (58.3%).	Grade 3/4 hand-foot syndrome (23.7%), oral mucositis (21.0%) and hypertension (7.9%). All hemorrhage were grade 1-2.

AST, aspertate aminotransferase; DCR, disease control rate; mPFS, median progression-free survival; mos, months; mOS, median overall survival; NPC, nasopharyngeal narcinoma; NR, not reached; ORR, objective response rate; SCCHN, squamous cell carcinoma of the head and neck.
