# Supplementary material for: Anlotinib as a third-line or further treatment for recurrent or metastatic nasopharyngeal carcinoma: a single-arm, phase 2 clinical trial
Source: BMC Med. 2023 Nov 7;21:423. doi: 10.1186/s12916-023-03140-x (PMC10631002; doi:10.1186/s12916-023-03140-x)
Supplement: Supplementary file 2 — Additional file 2. Study protocol. [file 12916_2023_3140_MOESM2_ESM.pdf]

Clinical Study Protocol

An Open-Label, Phase II Study of Anlotinib Treatment in Patients With Recurrent or Metastatic Nasopharyngeal Carcinoma after failure of no less than second-line chemotherapy or targeted therapy

Protocol Version: 3.0

Protocol Date: July 27, 2019

Sponsor: Sun Yat-Sen University Cancer Center

Study Director: Qingqing Cai

Table of Contents

SYNOPSIS OF PROTOCOL 4
1. STUDY BACKGROUND 6
1.1 Epidemiology of Nasopharyngeal Carcinoma and Current Treatment Modalities 6
1.2 Chemotherapy Regimens for Advanced Recurrent or Metastatic Nasopharyngeal Carcinoma 6
1.3 Study on Target for Anti-angiogenic Therapy in the Treatment of Nasopharyngeal Carcinoma 8
1.4 Mechanism of Action of Anlotinib 10
1.5 Clinical Efficacy of Anlotinib 10
2. STUDY OBJECTIVES AND ENDPOINTS 12
2.1 Primary Objective 12
2.2 Secondary Objectives 12
2.3 Study Endpoints 13
2.3.1 Primary endpoints 13
2.3.2 Secondary endpoints 13
3. STUDY POPULATION 13
3.1 Target Population 13
3.2 Total Planned Enrollment 13
3.3 Inclusion Criteria 14
3.4 Exclusion Criteria 15
3.5 Withdrawal Criteria 18
4. STUDY DRUG 18
4.1 Strength and Shelf Life of Study Drug 18
4.2 Storage of Study Drug 18
4.3 Route of Administration 19
4.4 Dose Adjustment 19
4.5 Concomitant Medications and Treatments 21
4.5.1 Medications that are prohibited or used with caution during the study 21
4.5.2 Concomitant medications allowed during the study 23
4.6 Adverse Reactions and Treatment 23
4.6.1 Hemorrhage 23
4.6.2 Thromboembolic events 24
4.6.3 Increased blood pressure 24
4.6.4 QT interval prolongation 26
4.6.5 Proteinuria and renal function abnormal 26
4.6.6 Thyroid insufficiency 27
4.6.7 Hand and foot skin reaction 27
4.6.8 Gastrointestinal adverse events 28
4.6.9 Hyperlipidaemia 28
4.6.10 Stomatitis 29
4.6.11 Reversible posterior leukoencephalopathy syndrome (RPLS) 29
5. STUDY PROCEDURES 29

5.1 Screening 29
5.2 Enrollment 31
5.3 Treatment Period 31
5.4 Follow-up 33
5.5 Early Termination of Study Visits 34
5.6 Unscheduled Visits 35
6. EFFICACY EVALUATION 35
6.1 Efficacy Evaluation Criteria 35
6.2 Efficacy Endpoints and Definitions 36
6.2.1 Efficacy endpoints 36
6.2.2 Definition of endpoints 36
6.3 Methods of Efficacy Evaluation 36
7. SAFETY EVALUATION 37
7.1 Adverse Events 37
7.2 Serious Adverse Events 37
7.3 Handling of Events 38
7.3.1 Adverse events 38
7.3.2 Serious adverse events 38
7.4 Documentation of Events 38
7.5 Reporting of Serious Adverse Events 38
8. DATA COLLECTION 39
9. STATISTICAL ANALYSIS 39
9.1 Overall Design 39
9.2 Analysis Population 39
9.3 Main Result Analysis 40
9.3.1 Statistics and analysis of baseline data 40
9.3.2 Safety analysis 40
9.3.3 Efficacy analysis 40
9.4 End of Study 41
10. SCHEDULE OF STUDY 41
11. QUALITY CONTROL AND QUALITY ASSURANCE 41
11.1 Quality Control 41
11.2 Quality Assurance 42
12. ETHICAL PRINCIPLES 42
13. DATA RETENTION AND SUMMARY 42
14. MAIN REFERENCES 43
15. APPENDIX 46
Appendix I Staging of Nasopharyngeal Carcinoma (AJCC/UICC 8th Edition) 46
Appendix II Response Evaluation Criteria in Solid Tumors (RECIST 1.1) 47
Appendix III NCI-CTCAE 4.0 Evaluation Criteria for Toxic Side Effects of Tumor Drugs 61
Appendix IV ECOG Scoring Criteria for Patient Quality of Life Evaluation 68
Appendix V FACT-H&N (V4) Quality of Life Questionnaire 69

SYNOPSIS OF PROTOCOL

Study Title	An Open-Label, Phase Ⅱ Study of Anlotinib Treatment in Patients With Recurrent or Metastatic Nasopharyngeal Carcinoma after failure of no less than second-line chemotherapy or targeted therapy
Study Objective	This is a single-arm study designed to evaluate the efficacy and safety of anlotinib hydrochloride in the treatment of patients with recurrent or metastatic nasopharyngeal carcinoma after failure of second-line or above chemotherapy or targeted therapy.
Study Design	Single-arm trial
Study Drug	Anlotinib hydrochloride
Study Population	Patients with recurrent or metastatic nasopharyngeal carcinoma after failure of second-line or above chemotherapy or targeted therapy
Sample Size	39
Treatment Regimen	Anlotinib hydrochloride will be administered orally at a dose of 12 mg once a day before breakfast, for 2 consecutive weeks, and discontinued for 1 week, i.e., three weeks (21 days) as a course of treatment. Treatment should continue until disease progression or occurrence of intolerable toxicity.
Study Period	Start date of enrollment: March 2019;<br>End date of enrollment: March 2021;<br>Expected date of last follow-up: March 2026.
Efficacy Endpoints	Disease control rate (DCR), overall response rate (ORR), progression free survival (PFS), overall survival (OS), complete remission (CR), partial remission (PR) and

	duration of response (DOR)
Safety Endpoints	Symptoms and signs, observed toxic reactions of chemotherapy, laboratory parameters, adverse events (AEs) and serious adverse events (SAEs)
Efficacy Evaluation	According to RECIST 1.1
Safety Evaluation	According to NCI-CTC AE 4.0
Quality of Life Evaluation	FACT-H&N (V4) Quality of Life questionnaire
Statistical Methods	In this single-arm study, the data will be analyzed mainly using descriptive statistics, with log-rank test for efficacy endpoint analysis and descriptive method and percentage for safety analysis.

1. STUDY BACKGROUND

1.1 Epidemiology of Nasopharyngeal Carcinoma and Current Treatment Modalities

Nasopharyngeal carcinoma (NPC) is a special type of malignant cancer of the head and neck with an extremely imbalanced geographical distribution. It is also referred to as "Canton tumor" due to a high incidence in Southern China. In 2012, there were 86,700 new cases of NPC reported globally, with the highest incidence in Southeast Asia[1]. The non-keratinizing pathological subtype accounts for more than 95% of NPC cases, which is highly sensitive to chemoradiotherapy. For patients with early-stage or locally advanced NPC, the standard treatment regimen is concurrent chemoradiotherapy[2], with 5-year survival rates of over 87%–96% for patients with early-stage NPC after treatment[3]. However, clinical studies showed that after the first course of treatment, 10%–36% of NPC patients still suffer from locally recurrent NPC, of which 65%–85% occur during the first 3 years after treatment[4]. In addition, about 15% of NPC patients have distant metastases at the time of diagnosis[5]. Patients with recurrent and/or metastatic NPC have a poor prognosis, with a median OS of only 20 months[6].

1.2 Chemotherapy Regimens for Advanced Recurrent or Metastatic Nasopharyngeal Carcinoma

At present, the first-line chemotherapy for recurrent or metastatic NPC has been gradually standardized, and platinum-based doublet chemotherapy regimens are widely used in clinical practice. The drugs used in combination chemotherapy include 5-fluorouracil (5-Fu), paclitaxel, docetaxel, gemcitabine and capecitabine. The most widely used combination chemotherapy regimen for patients with recurrent metastatic NPC has been the combination of 5-Fu with cisplatin (PF), but it has poor efficacy,

with an objective response rate of 40%–60% and a median OS of 15–20 months[7–10]. In addition, this regimen has the disadvantages of severe toxic and side effects such as stomatitis and inconvenient use. In a phase II clinical study by Chen et al.[11], the combination of paclitaxel with cisplatin (TP) as first-line treatment in patients with recurrence and metastasis showed a remission rate of 79%, PFS of 8.6 months and OS of 22.7 months. In another study, the combination of lobaplatin with docetaxel as first-line chemotherapy for recurrent and metastatic NPC showed an overall response rate of 61.5% and a PFS of 10.0 months[12]. In August 2016, Lancet published online the phase III clinical findings of Professor Zhang Li and his team on the combination of gemcitabine + cisplatin (GP) versus PF for the treatment of recurrent or metastatic NPC. The GP regimen showed a better objective response rate (64% vs. 42%), PFS (7.0 months vs. 5.6 months) and OS (29.1 months vs. 20.1 months), 45% risk reduction in disease progression, and 38% risk reduction in death, compared with the PF regimen, with a similar overall incidence of adverse events[13]. Unfortunately, 10% of the subjects treated with GP failed to achieve disease control (90% DCR), more than 30% of the subjects failed to achieve disease remission (64% ORR), and the median progression-free survival was only 7 months.

Although the efficacy of first-line chemotherapy for recurrent or metastatic nasopharyngeal carcinoma has been confirmed, a significant proportion of patients still experience disease progression after receiving first-line chemotherapy. There is no particularly effective treatment option available for patients failing first-line chemotherapy. Although second-line chemotherapy drugs such as gemcitabine and capecitabine have shown certain efficacy, they failed to result in a significant improvement in median survival, which was only 7–11 months[14]. It is difficult for NPC patients with distant metastasis to achieve a durable response. Most patients experience disease progression in a short period time after initial response, and the site of recurrence usually becomes resistant to chemotherapy. Therefore, there is an urgent need to develop novel therapeutic drugs to provide effective treatment options for patients with recurrent/metastatic NPC after the failure of two or more lines of chemotherapy or targeted therapy.

1.3 Study on Target for Anti-angiogenic Therapy in the Treatment of Nasopharyngeal Carcinoma

Tumors depend on angiogenesis for continuous replication and tumor growth. As early as in 1971, Folkman, an American scholar, firstly proposed that tumor growth was closely related to blood vessels and pointed out that tumors could be treated by blocking the growth of blood vessels. Vascular endothelial growth factor (VEGF), one of the most important angiogenic factors, can directly act on vascular endothelial cells to stimulate their mitosis, thus promoting the growth of new blood vessels. The angiogenesis pathway is one of the current focuses of drug research and development, and a number of drugs have been developed, including monoclonal antibodies and small molecule tyrosinase inhibitors (Table 1).

Table 1. Research progress on VEGF-targeted anti-angiogenic drugs

English Drug Name	Chinese Drug Name	Trade Name	Drug Type	Drug Sensitivity Testing	Remarks (Lung Cancer)	Indications (except Lung Cancer)
Bevacizumab	贝伐珠单抗	Avastin/安维汀	Monoclonal antibody	VEGF	First line (America, China)	Metastatic colorectal carcinoma, malignant glioma, metastatic renal cell carcinoma, cervix carcinoma, ovarian cancer
Ramucirumab	雷莫芦单抗	Cyramza	Monoclonal antibody	VEGFR2	Second line (America)	Colorectal carcinoma, gastric cancer
Nintedanib	尼达尼布	Ofev	Small molecule	VEGFR1-3, PDGFRα/β, FGFR1-3, etc.	Second line (Europe)	Pulmonary fibrosis
Endostatin	重组人血管内皮抑制素注射液	Endostar	Recombinant human endostatin	Suppressing tumor angiogenesis through inhibiting vascular endothelial cell migration	First/second line (China)	None
**Drugs under development**						
Anlotinib	安罗替尼		Small molecule	VEGFR1-3, PDGFRα/β, FGFR1-3, etc.	Third line (success)	None
Apatinib	阿帕替尼		Small molecule	VEGFR2	Third line (phase III in progress)	Gastric cancer
Sunitinib	舒尼替尼	Sutent	Small molecule	KIT, PDGFRβ, VEGFR1-3, etc.	Two or more lines (failure)	GIST, renal cell carcinoma, pancreatic neuroendocrine tumor
Sorafenib	索拉菲尼	Nexavar/多吉美	Small molecule	KIT, PDGFRβ, VEGFR1-3, etc.	Third line (failure)	Hepatocellular carcinoma, renal cell carcinoma, differentiated thyroid carcinoma
Fruquintinib	呋喹替尼		Small molecule	VEGFR1-3, FGFR1, KIT, etc.	First line (phase III in	None

Studies showed that overexpression of vascular endothelial growth factor-a (VEGF-A) associates with poor prognosis in head and neck squamous cell carcinoma (HNSCC). A meta-analysis of 1002 cases of HNSCC from 12 studies investigated the VEGF-A expression and found that the 2-year mortality rate in HNSCC subjects with VEGF-positive staining was twice that in negative subjects[15]. One study confirmed that VEGF plays a crucial role in the induction of angiogenesis and lymph node metastasis in NPC[16]. In another study, Qian et al. found significant increases in serum VEGF levels in 65 subjects with metastatic NPC[17]. The study results showed that 67% of NPC subjects showed VEGF overexpression, and VEGF expression was higher in subjects with EBV-positive tumors and was associated with increased recurrence rate, positive lymph node ratio, and decreased survival[18]. A preliminary study by Druzgal et al. demonstrated that serum cytokines and angiogenic factors can be used as markers for judging the efficacy in subjects with head and neck cancers before and after treatment[19]. At a mean follow-up of 37 months, subjects with decreased VEGF levels after treatment maintained longer disease remission than those with elevated VEGF levels after treatment. In this study, 7% of subjects were NPC patients.

Anti-angiogenic target also gradually becomes a hot topic in NPC clinical study. In a North American multicenter phase II study (RTOG 0615) reported in Lancet Oncology in 2012[20], bevacizumab plus chemoradiotherapy for locally advanced NPC (stage IIB–IVB) showed a 2-year OS of 90.9% and a 2-year PFS of 74.7%. No Grade 3–4 hemorrhage and Grade 5 adverse events occurred in this study, suggesting the efficacy and tolerability of bevacizumab plus chemoradiotherapy for locally advanced NPC, which is expected to improve patients' survival. In a study reported in Annals of Oncology in 2013 regarding the combination of sorafenib with PF as a second-line treatment in 54 patients with recurrent or metastatic NPC[21], the PFS and OS, as two primary study endpoints were 7.2 months and 11.8 months, respectively, and the major adverse reactions included hand and foot syndrome, myelosuppression and gastrointestinal reactions, which the patients could tolerate. This study demonstrated

the feasibility of sorafenib plus PF regimen for the treatment of recurrent or metastatic NPC. In a single-arm exploratory single-agent study of sunitinib[22] in 14 patients with recurrent or metastatic NPC after radiotherapy and multiple lines of chemotherapy, tumor shrinkage occurred in 5 out of the 10 patients who underwent at least one radiological evaluation, also suggesting the efficacy of sunitinib in patients with recurrent or metastatic NPC.

1.4 Mechanism of Action of Anlotinib

Anlotinib hydrochloride (AL3818) is a novel small molecule multi-target tyrosine kinase inhibitor, belonging to anti-angiogenic agents. Its principal mechanism of action is as follows: 1) Marked inhibitory activity against angiogenesis-related kinases, such as vascular endothelial growth factor receptor (VEGFR) 1/2/3, fibroblast growth factor receptor (FGFR) 1/2/3, and other tumor cell proliferation-related kinases, such as platelet-derived growth factor receptor (PDGFR) α/β, c-Kit and Ret. It can inhibit a broader spectrum of angiogenic kinases (e.g., Met, FGFR1/2/3). 2) Marked inhibitory activity against some kinase targets under studies, such as Aurora-B, c-FMS and DDR1. 3) Marked inhibitory activity against various kinase mutants, such as PDGFRα, cKit, Met and epidermal growth factor receptor (EGFR). The inhibitory activity against the mutants is even stronger than that against the wild type. This drug is developed independently in China and belongs to class 1.1 anti-cancer new drugs.
The maximum tolerated dose identified in the preliminary study was 12 mg qd in the dose-finding study. Through tolerance observation in 21 subjects successively, 12 mg qd for 2 consecutive weeks followed by 1-week discontinuation was generally recognized as the recommended regimen for subsequent studies, and 12 mg qd was the maximum tolerated dose.

1.5 Clinical Efficacy of Anlotinib

Several clinical studies have demonstrated the clinical efficacy of anlotinib. In a phase I clinical study of anlotinib in the treatment of multiple solid tumors including colon

adenocarcinoma, non-small cell lung cancer (NSCLC), clear cell renal cell carcinoma, medullary thyroid cancer and soft tissue sarcoma, 20 patients were evaluated for the antitumor activity of anlotinib, with PR in 3 cases, SD in 14 cases, and disease progression in 3 cases. The major adverse reactions included hypertension, increased triglycerides, hand and foot skin reaction, and increased lipase. This study showed that anlotinib had a good safety profile and broad-spectrum antitumor activity, providing a basis for further studies[23].

In a randomized controlled, double-blind, nationwide multicenter phase II clinical study (ALTER302) of anlotinib for the treatment of non-small cell lung cancer, the safety and efficacy of anlotinib as a third-line treatment in patients with advanced refractory NSCLC was evaluated. A total of 117 patients were enrolled in the study and were randomized to the placebo control group and the anlotinib group (12 mg qd). The study results showed a better PFS (4.8 months vs. 1.2 months), overall response rate (ORR, 10.0% vs. 0%) and mean OS (9.3 months vs. 6.3 months) in the anlotinib group than in the placebo group. In terms of safety, the incidence of Grade 3–4 adverse reactions in the anlotinib group was only 21.67%[24].

ALTER0303 was a multicenter, randomized, double-blind, placebo-controlled, parallel-group phase III clinical trial involving 437 patients with advanced metastatic NSCLC after the failure of at least two systemic chemotherapies (progression after corresponding targeted therapy in patients who were positive for both EGFR mutation and ALK). The primary endpoint of this study was OS, and the secondary endpoints included PFS and ORR. Patients were randomized (2:1) into two groups: the treatment group received a daily dose of 12 mg of anlotinib for 2 consecutive weeks followed by 1-week discontinuation (n = 294); the control group received placebo (n = 143). The baseline characteristics (i.e., sex, age, stage, ECOG score, histological type, gene mutation status, and prior treatment history) were comparable between the two groups. The primary efficacy endpoint was analyzed in 292 surviving patients (66.82%) after a median duration of treatment of 6 cycles in the anlotinib group and 2 cycles in the placebo group. The anlotinib regimen showed a better median OS (9.46 months vs. 6.37 months), a 30% risk reduction in death (hazard ratio = 0.70), and a

1-year survival rate (39.53% vs. 27.79%), compared with the placebo. In addition, the data also showed that the efficacy of anlotinib was not affected by the mutational status of the EGFR driver gene, i.e., anlotinib could exert a good treatment effect regardless of the presence or absence of EGFR mutation[25].

At present, the treatment effect of second-line chemotherapy for patients with recurrence and metastasis is not satisfactory. Therefore, there is an urgent need to develop effective drugs to provide new treatment options for patients who have failed two or more lines of chemotherapy or targeted therapy. The efficacy and safety of anlotinib have been demonstrated in clinical studies of other tumors, and other anti-angiogenic target agents have also demonstrated antitumor activity against NPC. This study aims to further investigate the efficacy and safety of anlotinib in the treatment of patients with recurrent or metastatic NPC after failure of two or more lines of chemotherapy, so as to provide new treatment options for recurrent and metastatic NPC.

2. STUDY OBJECTIVES AND ENDPOINTS

2.1 Primary Objective

To evaluate the efficacy and safety of anlotinib hydrochloride in the treatment of patients with recurrent or metastatic nasopharyngeal carcinoma after failure second-line or above chemotherapy or targeted therapy.

2.2 Secondary Objectives

1) To evaluate the long-term survival of patients.

2) To evaluate the quality of life of patients.

2.3 Study Endpoints

2.3.1 Primary endpoints

- DCR (perform efficacy evaluation according to RECIST v1.1; when the patient experiences objective remission for the first time, confirm after receiving two more courses of treatment)

2.3.2 Secondary endpoints

- ORR, PFS, OS, CR, PR and DOR (according to RECIST v1.1)
- Safety and adverse events (according to NCI CTC AE v4.0)
- Quality of life assessment (FACT-H&N.V4 Quality of Life questionnaire)

3. STUDY POPULATION

3.1 Target Population

Patients with recurrent or metastatic nasopharyngeal carcinoma after failure of second-line or above chemotherapy or targeted therapy

3.2 Total Planned Enrollment

A single-stage phase II design with a type I error of 5% and power of 80% was used to calculate the sample size. We considered the anlotinib treatment to be ineffective if DCR was ≤20% based on the result from the phase II study of gefitinib treatment for RM-NPC[27], and effective if DCR was ≥40% based on the DCR of 54.5% (95% CI, 38.0-70.2) achieved by pazopanib in RM-NPC patients[28]. An estimated sample size of 35 patients was required. If at least 11 cases of CR or PR or SD were observed, the drug would be deemed effective. Assuming a dropout rate of 10%, the maximum

estimated sample size was 39 patients.

3.3 Inclusion Criteria

3.3.1 Histopathological diagnosis of differentiated or undifferentiated locally recurrent or metastatic NPC (WHO Stage II-III);

3.3.2 Patients with clinical stage IVB NPC [Appendix I, AJCC staging system for nasopharyngeal cancer, 8th edition] after failure of first-line platinum-based chemotherapy (monotherapy or combination therapy) and second-line single-agent or combination chemotherapy or targeted therapy. Treatment failure is defined as progression during or after chemotherapy after recurrence/metastasis; progression within 6 months after concurrent chemoradiotherapy is considered first-line treatment. All regimen changes due to drug intolerance are not considered treatment failure;

3.3.3 No history of other malignant tumors, and having no other malignant tumors at the time of diagnosis;

3.3.4 Male or female patients aged 18–70 years;

3.3.5 ECOG performance status score of 0–2;

3.3.6 Estimated survival ≥ 3 months;

3.3.7 Non-lactating women and those of childbearing age must have a negative pregnancy test (serum or urine) within 14 days prior to enrollment, and be willing to take effective contraceptive measures during the study;

3.3.8 At least one measurable lesion per RECIST (≥ 10 mm in longest diameter by B-scan, MRI or spiral CT scan, and having not undergone radiotherapy);

3.3.9 Laboratory tests must meet the following criteria:

- Blood routine: Neutrophil count (ANC) ≥ 1.5 × 10^9/L, platelet count (PLT) ≥ 100 × 10^9/L, and hemoglobin (HGB) ≥ 90 g/L;
- Liver function: Total bilirubin ≤ 1.5 times the upper limit of normal (ULN); blood aspartate aminotransferase (AST) and blood alanine aminotransferase (ALT) ≤ 2.5× ULN (or ≤ 5× ULN for subjects with liver metastases); and alkaline phosphatase ≤ 5× ULN;

- Renal function: Serum creatinine (Cr) ≤ 1.5× ULN; and creatinine clearance ≥ 50 mL/min;
- Urinalysis: Urine protein < 2+ (subjects with baseline urine protein of ≥ 2+ should undergo a 24-hour urine protein test within 7 days and can be enrolled only when urine protein < 1 g);
- Coagulation function: INR and APTT ≤ 1.5× ULN;

3.3.10 No severe cardiopulmonary dysfunction;

3.3.11 Sign the informed consent form;

3.3.12 Ability to comply with the study protocol.

3.4 Exclusion Criteria

3.4.1 Known allergy to any drug used in the study;

3.4.2 Pregnant or breastfeeding women;

3.4.3 Participated in other clinical trial involving another drug within 4 weeks prior to the initiation of the study;

3.4.4 Prior treatment with bevacizumab or small molecule tyrosine kinase inhibitors of VEGFR (e.g., famitinib, sorafenib, sunitinib, regorafenib, apatinib and fruquintinib);

3.4.5 Recurrence of nasopharyngeal lesions after radiotherapy, followed by secondary radiotherapy;

3.4.6 Palliative radiotherapy for symptom control within 28 days prior to enrollment;

3.4.7 Systemic immunomodulatory therapies (including but not limited to interferon and interleukin-2) within 28 days prior to enrollment;

3.4.8 Tumor cell invasion in vital blood vessels (e.g., encasement of internal carotid artery/vein) by MRI; or the patient's tumor is very likely to affect vital blood vessels and cause fatal hemorrhage during treatment, as judged by investigators;

3.4.9 History of severe hemorrhage, and any other Grade ≥3 bleeding or hemorrhage per CTCAE 4.0 within 4 weeks prior to screening;

3.4.10 Coagulation abnormalities, and evidence or history of bleeding diathesis (INR

within normal range without the use of anticoagulants 14 days prior to signing the informed consent form); treatment with anticoagulants or vitamin K antagonists, such as warfarin, heparin or analogues thereof; on the premise of the prothrombin time as International Normalized Ratio (INR) ≤ 1.5, low-dose warfarin (1 mg orally, once a day) or low-dose aspirin (no more than 100 mg daily) is allowed for preventive purposes;

3.4.11 Unstable angina and/or congestive heart failure or vascular disease requiring hospitalization (e.g., aortic aneurysm or peripheral venous thrombosis requiring surgical repair), or other cardiac impairment that, as judged by investigators, may affect the safety evaluation of the study drug (e.g., poorly controlled arrhythmia, myocardial infarction or ischemia) within 12 months prior to enrollment;

3.4.12 Hypertension that is not well controlled with a single antihypertensive drug (defined as systolic blood pressure > 140 mmHg, and diastolic blood pressure > 90 mmHg); or combination therapy with two or more antihypertensive drugs; clinically significant (e.g., active) cardiovascular diseases - such as cerebrovascular accident (≤ 6 months prior to randomization), myocardial infarction (≤ 6 months prior to randomization), unstable angina, Class II congestive heart failure or above per New York Heart Association (NYHA), or severe arrhythmias that cannot be controlled with drugs or have potential impact on study treatment;

3.4.13 Esophageal and fundal gastric varices, active ulcers, intestinal perforation, or intestinal obstruction within 6 months prior to enrollment;

3.4.14 History of abdominal fistula, gastrointestinal perforation, intra-abdominal abscess, or acute gastrointestinal bleeding within 6 months prior to enrollment;

3.4.15 Multiple factors affecting oral administration and absorption of the study drug (e.g., inability to swallow, postoperative period following gastroenterectomy, chronic diarrhea and intestinal obstruction);

3.4.16 Occurrence of arterial/venous thrombosis events, Grade ≥3 venous thromboembolism per NCI CTCAE, such as cerebrovascular accident (including transient ischemic attacks), deep vein thrombosis (except for venous thrombosis triggered by intravenous catheterization during prior chemotherapy that has been

cured as judged by investigators) and pulmonary embolism within 6 months prior to enrollment;

3.4.17 Objective evidence of pulmonary fibrosis, interstitial pneumonia, pneumoconiosis, radiation pneumonitis, drug-related pneumonia and severely impaired lung function, etc. in the past and at present;

3.4.18 Exacerbation of chronic obstructive pulmonary disease (COPD) or other respiratory diseases requiring hospitalization within 28 days prior to enrollment;

3.4.19 Active pulmonary infection and/or acute bacterial or fungal infection requiring intravenous antibiotic treatment within 28 days;

3.4.20 Obvious jaundice due to abnormal liver function within 7 days;

3.4.21 Renal insufficiency: Urinalysis shows proteinuria > 2+ and 24-hour urine protein test demonstrates urine protein > 1.0 g;

3.4.22 Minor surgery (including catheterization, excluding peripherally inserted central catheterization) within 2 days prior to enrollment;

3.4.23 Major surgery within 28 days prior to enrollment;

3.4.24 Treatment with a strong CYP3A4 inhibitor within 1 week prior to enrollment or treatment with a strong CYP3A4 inducer within 2 weeks prior to enrollment;

3.4.25 Chronic non-healing wounds or incompletely healed fractures;

3.4.26 Symptomatic metastases to the central nervous system (e.g., cerebral edema requiring hormonal intervention, or progression of brain metastasis) (proven or suspected);

3.4.27 Severe or uncontrolled infection;

3.4.28 History of psychotropic drug abuse and unable to quit, or history of mental disorders;

3.4.29 History of immunodeficiency, including positive HIV result, or other acquired and congenital immunodeficiency diseases, or history of organ transplantation;

3.4.30 Virological tests during the screening period show either of the following: Positive HBsAg result and HBV DNA titer in the peripheral blood $\geq 1 \times 10^3$ copies/L; positive anti-HCV result;

3.4.31 History of other malignant tumors in the past 5 years, except cured skin basal

cell carcinoma, cervical carcinoma in situ and superficial bladder cancer;

3.4.32 Concomitant diseases that may severely impair the safety of patients or make the patients unable to complete the study, as judged by investigators.

3.5 Withdrawal Criteria

3.5.1 Subjects voluntarily withdraw the informed consent and request to withdraw from the clinical trial;

3.5.2 Disease progression with medical radiological evidence (e.g., CT, MRI, B-scan examination, etc.) or clinical progression clearly judged by investigators (the reasons for judging progression should be recorded in detail);

3.5.3 Pregnancy in female subject occurs during the study;

3.5.4 Other conditions that necessitate the withdrawal from the study, as considered by investigators (the withdrawal reasons should be recorded in detail);

3.5.5 The sponsor terminates the study due to safety concern or other special reasons.

4. STUDY DRUG

4.1 Strength and Shelf Life of Study Drug

Name of Drug	Characters	Strength	Directions for Use	Shelf Life
**Anlotinib Hydrochloride Capsules**	The contents are white or off-white powder or granules	12 mg, 10 mg, 8 mg	Oral	18 months

4.2 Storage of Study Drug

Store sealed under 25°C and away from light

4.3 Route of Administration

Based on the human pharmacokinetic studies, 12 mg qd for 2 consecutive weeks followed by 1-week discontinuation was generally recognized as the recommended regimen for subsequent studies, and 12 mg qd was the maximum tolerated dose. In addition, the study results showed that the t_{max} of anlotinib in humans was increased with a high-fat diet as compared to administration under fasting conditions, and drug absorption was slightly reduced (approximately 80% that of administration under fasting conditions). Therefore, anlotinib is recommended to be administered under fasting conditions in subsequent clinical practice.

Anlotinib hydrochloride will be administered orally to all enrolled patients at a dose of 12 mg qd before breakfast, for 2 consecutive weeks, and discontinued for 1 week, i.e., three weeks (21 days) as a course of treatment. Treatment should continue until disease progression or occurrence of intolerable adverse reactions.

4.4 Dose Adjustment

Adverse reactions should be closely monitored during the administration of the study drug, and the dose should be adjusted according to the patient's tolerance. Adverse reactions caused by the study drug can be treated by symptomatic treatment, drug interruption and/or dose adjustment. The dose should be adjusted under the guidance of a physician according to the severity of adverse reactions: 1) First dose adjustment: 10 mg qd for 2 weeks, followed by 1-week discontinuation; 2) Second dose adjustment: 8 mg qd for 2 weeks, followed by 1-week discontinuation. If the dose of 8 mg remains intolerable, the drug should be permanently discontinued.

Dose adjustments for each adverse reaction are as follows:

- **Hematologic Toxicities**

(1) Platelet count decreased

**Grade of AE**	**Treatment Recommendations**	**Dose Adjustment**
Grade 1	Perform visits as scheduled	Maintain the original dose

Grade 2	Delay dosing until AE resolves to ≤ grade 1	Maintain the original dose
Grade 3	Delay dosing and provide medical interventions until AE resolves to ≤ grade 1	Continue at a lower dose
Grade 4	Permanent discontinuation	/

(2) Neutrophil count decreased

**Grade of AE**	**Treatment Recommendations**	**Dose Adjustment**
Grade 1	Perform visits as scheduled	Maintain the original dose
Grade 2	Delay dosing until AE resolves to ≤ grade 1	Maintain the original dose
Grade 3-4	Delay dosing and provide medical interventions until AE resolves to ≤ grade 1	Continue at a lower dose

- **Non-Hematologic Toxicities**

(1) Hepatic function abnormal (elevated ALT, AST or TBIL)

**Grade of AE**	**Treatment Recommendations**	**Dose Adjustment**
Grade 1	Perform visits as scheduled	Maintain the original dose
Grade 2	Delay dosing until AE resolves to ≤ grade 1	Maintain the original dose
Grade 3-4	Delay dosing and provide medical interventions until AE resolves to ≤ grade 1	Continue at a lower dose

(2) Proteinuria

**Grade of AE**	**Definition**	**Treatment Recommendations**	**Dose Adjustment**
Grade 1	1+ proteinuria, or urine protein < 1.0 g/24 h	Perform visits as scheduled	Maintain the original dose
Grade 2	2+ proteinuria, and urine protein < 1.0 g/24 h	Perform visits as scheduled	Maintain the original dose
	2+ proteinuria, and urine protein is 1.0–3.4 g/24 h	Delay dosing and provide medical interventions until proteinuria ≤ 2+ and urine protein < 1.0 g/24 h	Maintain the original dose
Grade 3-4	Urine protein ≥ 3.5 g/24 h;	Delay dosing and provide medical interventions until proteinuria ≤ 2+ and urine protein < 1.0 g/24 h	Continue at a lower dose

(3) Hemorrhage events (including hemoptysis, digestive tract hemorrhage, nasal hemorrhage, bronchial hemorrhage, gingival hemorrhage, gross hematuria, fecal occult blood, and cerebral hemorrhage)

Grade of AE	Treatment Recommendations	Dose Adjustment
Grade 1	Perform visits as scheduled	Maintain the original dose
Grade 2	Delay dosing until AE resolves to ≤ grade 1	Continue at a lower dose; if event recurs, consider permanent discontinuation
≥ Grade 3	Permanent discontinuation	/

(4) Hypertension

Grade of AE	Treatment Recommendations	Dose Adjustment
Grade 1-2	Perform visits as scheduled	Maintain the original dose
Grade 3	Delay dosing until AE resolves to ≤ grade 2	Continue at a lower dose
Grade 4	Permanent discontinuation	/

(5) Hand and foot skin reaction

Grade of AE	Treatment Recommendations	Dose Adjustment
Grade 1-2	Perform visits as scheduled	Maintain the original dose
Grade 3-4	Delay dosing until AE resolves to ≤ grade 2	Continue at a lower dose

(6) Others

Grade of AE	Treatment Recommendations	Dose Adjustment
Grade 1	Perform visits as scheduled	Maintain the original dose
Grade 2–3	Delay dosing until AE resolves to ≤ grade 1	Continue at a lower dose
Grade 4	Permanent discontinuation	/

4.5 Concomitant Medications and Treatments

4.5.1 Medications that are prohibited or used with caution during the study

4.5.1.1 Prohibited medications

CFDA-approved anticancer medications are prohibited during the study, including chemotherapy agents, modern Chinese medicine preparations, and immunomodulators (such as thymosin, interferon, interleukin-2, Zilongjin, and lentinan).

4.5.1.2 Medications used with caution

(1) Anticoagulants or antithrombotic medications:

Mainly including but not limited to salicylic acid derivatives, such as aspirin;

Heparins, such as low molecular weight heparin, enoxaparin, dalteparin, and ardeparin; Prophylactic anticoagulants after cardiovascular and cerebrovascular events, such as clopidogrel and ticagrelor.

(2) Medications interfering with liver P450 enzymes:

Including but not limited to CYP3A inducers (carbamazepine, rifampicin, and phenobarbital) and inhibitors (ketoconazole, itraconazole, erythromycin, and clarithromycin). The following medications should be used with caution: CYP3A4 substrates (simvastatin, cyclosporine, and pimozide); other medications metabolized by CYP3A4 (such as benzodiazepine, dihydropyridine, calcium ion antagonists (which can be selected as appropriate for hypertension that cannot be controlled by ACEIs) and HMG-COA reductase inhibitors; CYP2C9 substrates (diclofenac, phenytoin, piroxicam, S-warfarin, and tolbutamide), and CYP2C19 substrates (diazepam, imipramine, lansoprazole, and S-mephenytoin).

(3) Medications that prolong QT interval:

Since anlotinib has the toxic side effect of prolonging the QT interval in clinical practice, medications that can prolong the QT interval must be used with caution during the study. These medications mainly include but are not limited to:

- Antimicrobials (clarithromycin, azithromycin, erythromycin, roxithromycin, metronidazole, moxifloxacin);
- Antiarrhythmic medications (quinidine, sotalol, amiodarone, disopyramide, procainamide);
- Antipsychotics (risperidone, fluphenazine, droperidol, haloperidol, thioridazine, pimozide, olanzapine, clozapine);
- Antifungals (fluconazole, ketoconazole);
- Antimalarials (mefloquine, chloroquine);
- Antidepressants (amitriptyline, imipramine, clomipramine, dothiepin, doxepin).

(4) Citrus, star fruit, grapefruit and grapefruit juice;

4.5.2 Concomitant medications allowed during the study

Subjects can receive supportive treatments. Supportive treatments can be combined with the following medications or related treatments: Antibiotics, analgesics, hormones, psychotherapy, or any other symptomatic treatment necessary to provide optimal supportive treatments. Other investigational antineoplastic agents or antineoplastic chemotherapy/endocrine therapy/immunotherapy are not within the scope of supportive treatment defined in the protocol.

Non-conventional treatments (e.g., herbal products or acupuncture) and vitamin/mineral supplementation are permitted if the investigator believes that they will not affect the study endpoints. Subjects may receive bisphosphonates for bone metastases during treatment.

Palliative radiation therapy involving a small area (irradiated areas must be < 5% of the marrow area) is permitted if systemic therapy or local analgesia fails to control painful bone metastases, provided that the radiation field does not include the target lesion.

During treatment, granulocyte colony-stimulating factor (G-CSF) and other hematopoietic growth factors may be used if clinical manifestations suggest or at the discretion of the investigator that treatment of acute toxicities such as febrile neutropenia is warranted. Subjects are allowed to use erythropoietin on a long-term basis.

4.6 Adverse Reactions and Treatment

4.6.1 Hemorrhage

VEGFR inhibitors can increase the risk of hemorrhage. Clinical studies showed that anlotinib increased the incidence of hemorrhage events compared with placebo. Therefore, clinicians should pay close attention to relevant symptoms and closely monitor the prothrombin time and international normalized ratio (INR) during dosing.

Once a grade 2 hemorrhage event is observed, treatment should be interrupted; if the severity of the event improves to < grade 2 within 2 weeks, treatment should be continued at a lower dose. Permanent discontinuation is required in case of hemorrhage events of grade 3 or higher.

Active symptomatic treatments should be provided for digestive tract hemorrhage, including fecal occult blood ≥ ++, hematemesis or bloody stool. Patients with upper gastrointestinal hemorrhage should be fasted and given acid suppression agents, medications for gastric mucosa protection, hemostasis (e.g., tranexamic acid, reptilase), blood transfusion and supportive treatments. Patients with lower gastrointestinal hemorrhage should be given hemostasis, blood transfusion and supportive treatments. Patients with unmanageable hemorrhage should be treated with surgical assistance.

4.6.2 Thromboembolic events

Clinical studies showed that anlotinib may increase the risk of thromboembolic events. Thrombosis-related adverse reactions should be closely monitored during dosing, and treatment interruption is recommended if such ARs are observed. If the AR recurs after treatment resumption, discontinuation is recommended.

4.6.3 Increased blood pressure

Increased blood pressure is the most common adverse reaction of VEGFR inhibitors. Previous clinical studies have shown that the incidence of hypertension events was high (about 13.61% for grade 3 or higher) in patients receiving Anlotinib Hydrochloride Capsules. Therefore, blood pressure should be measured daily for the first 6 weeks of dosing and 2–3 times a week during the subsequent dosing period.

In case of hypertension, the investigators are recommended to handle the event as follows:

- Grade 1: Prehypertension (SBP 120–139 mmHg or DBP 80–89 mmHg): Lack of indications for antihypertensive drugs, only blood pressure

monitoring is required;

- Grade 2: Stage I hypertension (SBP 140–159 mmHg or DBP 90–99 mmHg): Requires medical interventions; Repeated or persistent (≥ 24 h), symptomatic SBP increases by > 20 mmHg or increases to > 140/90 mmHg compared to previous normal range; Requires monotherapy and blood pressure monitoring; Thiazide diuretics are commonly used, and angiotensin-converting enzyme inhibitors (ACEIs), angiotensin II receptor blockers (ARBs), β-blockers, and calcium channel blockers may also be considered;
- Grade 3: Stage II hypertension (SBP ≥ 160 mmHg or DBP ≥ 100 mmHg): Requires medical intervention; Requires multi-drug therapy, usually thiazide diuretics combined with ACEIs, β-blockers or calcium channel blockers;
- Grade 4: Life-threatening (e.g., malignant hypertension, transient or permanent neurologic deficit, hypertension emergency): Requires urgent intervention;

  If grade 3/4 hypertension (SBP ≥ 180 mmHg or DBP ≥ 110 mmHg) is observed, anlotinib should be interrupted; If grade 3/4 hypertension is observed after anlotinib resumption, treatment should be continued at a lower dose. If grade 3/4 hypertension persists, discontinuation is recommended.
- Hypertension emergencies are defined as having DBP > 120 mmHg accompanied by acute or progressive target organ damage (such as brain infarction, intracranial or subarachnoid hemorrhage, and hypertensive encephalopathy). Progressive or accelerated hypertension on the basis of chronic primary hypertension is the most common (about 40% to 50%) hypertension emergency. Treatment should be discontinued once a hypertension emergency is observed. The subject should withdraw from the clinical study and receive specialized cardiovascular treatment in time.

4.6.4 QT interval prolongation

Anlotinib can prolong the QT/QTc interval, which may increase the risk of ventricular tachyarrhythmias (e.g., torsades de pointes) or sudden death. Therefore, patients with congestive heart failure, electrolyte abnormality, or receiving medications known to prolong the QTc interval should undergo regular (every 3–6 weeks) electrocardiogram and electrolyte (sodium, magnesium, potassium, and calcium) tests. If the results of two consecutive independent ECG show QTc interval > 500 ms, anlotinib should be interrupted until QTc interval is ≤ 480 ms or returns to baseline level (if QTc interval at baseline is > 480 ms), then the administration can be continued at a lower dose with close ECG monitoring.

For patients with any grade of QTc prolongation (≥ 450 ms) accompanied by torsades de pointes, polymorphic ventricular tachycardia, symptoms or signs of severe arrhythmia, permanent discontinuation of the study drug and specialized cardiovascular treatment are recommended.

Patients with underlying cardiac dysfunction should undergo echocardiography every 6 weeks, and discontinuation is recommended for patients with a left ventricular ejection fraction < 50%.

4.6.5 Proteinuria and renal function abnormal

Proteinuria is one of the common adverse reactions of VEGFR inhibitors. Therefore, urinalysis should be performed weekly for patients throughout the treatment. Patients with urine protein ≥ ++ for 2 consecutive tests should undergo a 24-hour urine protein test. Measures including treatment interruption, dose adjustment and permanent discontinuation are recommended according to the severity of adverse reactions (Table 1).

Recommended drugs: Compound Nephritis Tablets. ACEIs and ARBs can be provided as they can lower renal tubular pressure and thereby alleviate proteinuria and reduce the risk of potential adverse cardiac events.

4.6.6 Thyroid insufficiency

In the ALTER0303 study, hypothyroidism was observed in 58 patients (19.39%) of the anlotinib group, including 1 patient (0.34%) with grade 3 hypothyroidism. Grade 1 hypothyroidism was observed in 4 patients (2.80%) of the placebo group. Thyroid function should be tested prior to the first dose and closely monitored throughout the treatment for all subjects. All subjects receiving anlotinib should be closely monitored for symptoms and signs of decreased thyroid function, including cold intolerance, decreased appetite, and edema. Patients with underlying hypothyroidism or hyperthyroidism should be given appropriate standard treatment when receiving anlotinib. Thyroid function [thyroid stimulating hormone (TSH), triiodothyronine (T3), tetraiodothyronine (T4)] should be tested every 3–6 weeks for patients with symptoms and signs of thyroid insufficiency. When TSH $\geq$ 20 mU/L or T3, T4, FT3 or FT4 is lower than the normal value, symptomatic treatment (e.g., Euthyrox) should be given as soon as possible.

4.6.7 Hand and foot skin reaction

Hand and foot skin reaction is characterized by redness, obvious discomfort, swelling, and tingling sensation in the palms or soles. It is one of the common clinical adverse reactions of VEGFR inhibitors.

Clinical manifestations of hand and foot skin reaction: Grade 1: Minor painless skin changes or dermatitis (e.g., erythema, edema, or hyperkeratosis); Grade 2: Painful skin changes (e.g., peeling, blistering, bleeding, swelling, or hyperkeratosis) affecting instrumental activities of daily living; Grade 3: Painful severe skin changes (peeling, blistering, bleeding, edema, or hyperkeratosis) affecting personal activities of daily living.

Patients with grade 1 hand and foot skin reaction should continue to be observed. Patients with grade 2 hand and foot skin reaction should be treated symptomatically, including strengthening skin care, keeping the skin clean, avoiding secondary

infections, compression and friction, topical use of lotions or lubricants containing urea and corticosteroids, and topical use of antifungal agents or antibiotics in case of infections under the guidance of a dermatologist.

Patients with grade ≥ 3 hand and foot skin reaction should continue treatment at a lower dose. Discontinuation is recommended if the adverse reaction persists.

4.6.8 Gastrointestinal adverse events

In the ALTER0303 clinical study, diarrhea was the most frequently reported treatment-related gastrointestinal AE. Diarrhea was reported by 86 patients (29.25%) in the anlotinib group, including 3 patients (1.02%) with grade 3 diarrhea. Other gastrointestinal AEs included oropharyngeal pain, stomatitis, vomiting, nausea, and abdominal pain. Supportive care for gastrointestinal AEs requiring treatment may include oral care, antiemetic and antidiarrheal treatments.

Supportive treatments may be given for patients experiencing grade 1–2 diarrhea throughout study, such as symptomatic treatment (e.g., 4 mg oral loperamide, then 2 mg every 2 hours until diarrhea resolves) at the time of onset. If grade 3/4 diarrhea is observed, anlotinib should be interrupted. If grade 3/4 diarrhea is observed again after anlotinib resumption, treatment should be continued at a lower dose. If the adverse reaction persists, discontinuation is recommended.

4.6.9 Hyperlipidaemia

The ALTER0303 study showed that anlotinib resulted in increased triglycerides and cholesterol. Hence, patients with hyperlipidemia are recommended to change to a low-fat diet. Hypercholesterolemia (27.75 mmol/L) or hypertriglyceridemia ($\geq 2.5\times$ ULN) of grade 2 or higher should be treated with hydroxymethylglutaryl coenzyme A (HMG-CoA) reductase inhibitors (e.g., atorvastatin) or appropriate lipid-lowering drugs.

4.6.10 Stomatitis

Stomatitis is one of the common adverse reactions of VEGFR inhibitors. For patients with stomatitis, Houfengsan, Kangfuxin and mouthwash are recommended.

4.6.11 Reversible posterior leukoencephalopathy syndrome (RPLS)

Reversible posterior leukoencephalopathy syndrome (RPLS) has been reported in cancer patients treated with VEGFR inhibitors and can be fatal. RPLS is a neurological disorder that may be accompanied by headache, seizures, somnolence, confusion, blindness, other visual and neurological disturbances, and mild to severe hypertension. MRI is the best established diagnostic method for RPLS. No such events have been reported in anlotinib studies, and relevant symptoms and signs should be closely monitored during practice. Permanent discontinuation is recommended for patients with RPLS.

5. STUDY PROCEDURES

Patients must read and sign the informed consent form (ICF) approved by the Ethics Committee (EC) prior to the study. The examinations for subjects and test procedures should be conducted according to the schedule of the clinical study, irrespective of the duration of drug discontinuation. Changes due to holidays, festivals and other administrative reasons are allowed within the time window of each examination.

5.1 Screening

The screening period begins with the signing of ICF and ends with the administration of the study drug or screening failure. Subjects must sign the ICF before undertaking the screening procedures specified in this study. Results from laboratory tests and radiological assessments required for routine clinical care prior to ICF signing may be used if they are within the specified time window.

Unless otherwise specified, the following procedures should be completed within 28 days prior to the administration of the study drug.

- ➢ Signing the informed consent form;
- ➢ Collection of demographics: Sex, date of birth, ethnicity, height, body weight;
- ➢ Tumor diagnosis: Primary tumor site, date of pathological diagnosis, pathological grade and metastatic site;
- ➢ History of tumor treatment
    - ✓ History of tumor surgery: Name of surgery, date of surgery, postoperative TNM stage, date of postoperative recurrence;
    - ✓ History of radiotherapy: Site of radiotherapy, dose, start and end dates;
    - ✓ History of neoadjuvant chemotherapy: Chemotherapy regimen, cycle, start and end time;
    - ✓ History of adjuvant chemotherapy: Chemotherapy regimen, cycle, start and end time;
    - ✓ Salvage therapy: Regimen, cycle, start and end time, best response, time to tumor progression, treatment change due to tumor progression;
    - ✓ History of comorbidities, past medications, and drug allergy;
    - ✓ Virology tests (within 14 days prior to the first dose): HbsAg (if positive, HBV-DNA test is required), HBsAb, HBeAg, HBeAb, HBcAb, HCV-Ab (if positive, HCV-RNA test is required), HIV-Ab, EBV-DNA;

The following procedures and a pregnancy test should be completed within 7 days and 72 hours prior to the administration of the study drug, respectively:

- ✓ ECOG score;
- ✓ Vital signs: Pulse, respiratory rate, body temperature, blood pressure;
- ✓ Comprehensive physical examination: General condition, head and face, skin, lymph nodes, neck, eyes, ENT, oral cavity, respiratory system, cardiovascular system, abdomen, reproductive-urinary system, musculoskeletal system, nervous system, mental status, and others;
- ✓ Blood routine: Red blood cell count, hemoglobin, platelet count, white blood cell count, neutrophil count, lymphocyte count;

- ✓ Urinalysis: White blood cell count, red blood cell count, urine protein. If urine protein is ≥ 2+, a 24-hour urine protein test is required;
- ✓ Fecal occult blood;
- ✓ Clinical chemistry: ALT, AST, GGT, total bilirubin, direct bilirubin, AKP, blood urea nitrogen or urea (blood urea nitrogen is preferred), total protein, albumin, creatinine, blood glucose, lactate dehydrogenase, K+, Na+, Ca2+, Mg2+, Cl-;
- ✓ Thyroid function: TSH, FT3, FT4;
- ✓ Coagulation function: APTT, PT, FIB, INR;
- ✓ Echocardiogram: LVEF assessment is included. Tests as clinically indicated;
- ✓ 12-lead ECG: Additional necessary tests should be performed according to the judgment of the investigators in case of any abnormality;
- ✓ Pregnancy test: Serum or urine (applicable to women of childbearing age);
- ✓ Radiological examination: CT or MRI of the nasopharynx, neck, chest, upper and lower abdomen (including the pelvis). Brain MRI (if MRI is contraindicated, CT can be used instead) should be performed when brain metastasis is suspected and confirmed. A bone scan should only be performed when clinically indicated and must be performed within 42 days before the first dose. During the screening period, tumor assessment can be completed within 4 weeks before administration, and radiological results obtained before signing the ICF can be used for tumor assessment as long as they meet the RECIST 1.1;
- ✓ AEs: AEs are recorded from the signing of ICF.

5.2 Enrollment

- **Confirmation of eligibility.**
- **Dosing.**

5.3 Treatment Period

- The following assessments should be completed before each dose in each cycle. If the test is completed within 7 days before the first dose during the screening

period, it is not required prior to the first dose.

- ✓ ECOG score;
- ✓ Vital signs: Pulse, respiratory rate, body temperature, blood pressure;
- ✓ Physical examination: General conditions, head and face, skin, lymph nodes, neck, eyes, ENT, oral cavity, respiratory system, cardiovascular system, abdomen, reproductive-urinary system, musculoskeletal system, nervous system, mental status, and others;
- ✓ Blood routine: Complete blood count and differential count (white blood cells, red blood cells, lymphocytes, monocytes, neutrophils, basophils, eosinophils, hemoglobin), platelet count;
- ✓ Clinical chemistry: ALT, AST, GGT, total bilirubin, direct bilirubin, AKP, blood urea nitrogen or urea (blood urea nitrogen is preferred), total protein, albumin, creatinine, blood glucose, lactate dehydrogenase, K+, Na+, Ca2+, Mg2+, Cl-;
- ✓ EBV-DNA test;
- ✓ Urinalysis: White blood cell count, red blood cell count, urine protein. If urine protein is ≥ 2+, a 24-hour urine protein test is required;
- ✓ Routine stool test: White blood cells, red blood cells, occult blood;
- ✓ Coagulation function: Prothrombin time, activated partial thromboplastin time, thrombin time, fibrinogen, D-dimer, international normalized ratio;
- ✓ Thyroid function: TSH, FT3, FT4 (FT3 and FT4 can be replaced by T3 and T4 if they are not available);
- ✓ 12-lead electrocardiogram (12-Lead ECG): Additional necessary tests should be performed according to the judgment of the investigators in case of any abnormality;
- ✓ Concomitant medication: Concomitant medication should be recorded in detail;
- ✓ AEs: AEs should be recorded in detail;
- ▪ Radiological examination (completed before dosing every two cycles): CT or MRI of the nasopharynx, neck, chest, and abdomen (including the pelvis). An enhanced scan is preferred if not contraindicated. The radiological examination should be performed every 6 weeks. For bone metastases, a bone scan is

performed only if the response for other lesions is assessed as CR and it is necessary to confirm whether bone metastases have completely disappeared or there are clinical indications. Subjects who withdraw from the study for any reason should undergo radiological examination at the time of withdrawal if no such examination has been performed within 3 weeks. Conditions for radiological examination should be the same as those at baseline (including scan slice thickness and contrast agent). A time window of ± 7 days is permitted for radiological examination, and unscheduled radiological examination can be performed when PD is suspected (e.g., symptomatic deterioration). In addition to radiologically confirmed PD, subjects who discontinue study treatment for other reasons should also undergo radiological examination every 6 weeks until documented PD, initiation of new antitumor therapy, loss to follow-up, or death. The time of radiological examination will not be adjusted for the delayed dosing period.

5.4 Follow-up

On-site follow-ups will be conducted 30 days after the last dose at the study site, and safety information (including AE outcome, new SAEs, and adverse events of special interest) will be collected by telephone follow-ups at 60 and 90 days after the last dose, with a time window of ± 7 days.

If a subject starts a new antitumor therapy within 30 days after the last dose, the follow-up should be completed before the new antitumor therapy.

- ✓ Blood routine: Red blood cell count, hemoglobin, platelet count, white blood cell count, neutrophil count, lymphocyte count;
- ✓ Clinical chemistry: ALT, AST, GGT, total bilirubin, direct bilirubin, AKP, blood urea nitrogen or urea (blood urea nitrogen is preferred), total protein, albumin, creatinine, blood glucose, lactate dehydrogenase, K+, Na+, Ca2+, Mg2+, Cl-;
- ✓ Thyroid function: TSH, FT3, FT4;
- ✓ AEs: AEs should be recorded in detail;

Concomitant medication: Concomitant medications are recorded until 90 days after the last dose. Concomitant medications for study drug-related AEs are recorded until 30 days after the last dose.

Survival follow-up will be performed by telephone and other effective methods once a month from the last dose to determine if the subject has initiated new antitumor therapy subsequently. If so, the treatment regimen, as well as start and end time will be recorded and the survival follow-up form will be completed.

For subjects who withdraw from the study due to "non-PD" (such as intolerable AEs), tumor assessment is recommended to be performed according to the frequency of efficacy evaluation specified in this study (every 6 weeks ± 7 days) during the tumor progression follow-up until PD, death or initiation of new antitumor therapy. The follow-up information should be recorded in the CRF.

5.5 Early Termination of Study Visits

If the subject has not undergone relevant assessments and examinations within 7 days prior to withdrawal, the following should be performed:

- ✓ ECOG score;
- ✓ Vital signs: Pulse, respiratory rate, body temperature, blood pressure;
- ✓ Comprehensive physical examination: General condition, head and face, skin, lymph nodes, neck, eyes, ENT, oral cavity, respiratory system, cardiovascular system, abdomen, reproductive-urinary system, musculoskeletal system, nervous system, mental status, and others;
- ✓ Blood routine: Red blood cell count, hemoglobin, platelet count, white blood cell count, neutrophil count, lymphocyte count;
- ✓ Urinalysis: White blood cell count, red blood cell count, urine protein. If urine protein is ≥ 2+, a 24-hour urine protein test is required;
- ✓ Clinical chemistry: ALT, AST, GGT, total bilirubin, direct bilirubin, AKP, blood urea nitrogen or urea (blood urea nitrogen is preferred), total protein, albumin, creatinine, blood glucose, lactate dehydrogenase, K+, Na+, Ca2+, Mg2+, Cl-;

- ✓ Thyroid function: TSH, FT3, FT4;
- ✓ EBV-DNA test;
- ✓ ECG;
- ✓ Radiological examination: Subjects who withdraw from the study should undergo a radiological examination at the end of treatment or at the time of withdrawal if no such examination has been performed within 3 weeks before withdrawal. In addition to radiologically confirmed PD, subjects who discontinue study treatment for other reasons should also undergo radiological examination every 6 weeks until documented PD, initiation of new antitumor therapy or death.
- ✓ AEs: AEs should be recorded in detail;
- ✓ Concomitant medication: Concomitant medication should be recorded in detail.

5.6 Unscheduled Visits

During the study, if unscheduled follow-up is required due to AE occurring within 90 days after the last dose or within 90 days before a new antitumor therapy, the following should be recorded:

- ✓ Concomitant medications;
- ✓ Adverse events;
- ✓ All relevant examinations performed (including radiological examination, if any).

6. EFFICACY EVALUATION

6.1 Efficacy Evaluation Criteria

Efficacy is evaluated according to the European Response Evaluation Criteria in Solid Tumors (RECIST 1.1) (see Attached Appendix II).

6.2 Efficacy Endpoints and Definitions

6.2.1 Efficacy endpoints

Efficacy endpoints mainly include disease control rate (DCR), progression free survival (PFS), overall survival (OS), overall response rate (ORR) and duration of response (DOR).

6.2.2 Definition of endpoints

Disease control rate (DCR) is defined as the percent of subjects with complete response (CR), partial response (PR) or stable disease (SD) as the best overall response;

Overall response rate (ORR) is defined as the percent of subjects with CR or PR as the BOR;

Progression free survival (PFS) is defined as the time from enrollment to PD or death due to any cause. For subjects with unknown disease status, PFS refers to the time from enrollment to last the follow-up;

Overall survival (OS) is defined as the time from enrollment to death due to any cause. If it is unclear whether the subject has died, OS refers to the time from enrollment to the last follow-up;

Complete remission (CR) is defined as the percent of subjects with CR as BOR.

Partial remission (PR) is defined as the percent of subjects with PR as BOR.

Duration of response (DOR) is defined as the time from the first documented response (CR or PR) to progressive disease (PD) or recurrence.

6.3 Methods of Efficacy Evaluation

Efficacy is primarily evaluated by MRI, CT, or nasopharyngoscopy at the site of lesion. Refer to RECIST 1.1 for specific evaluation criteria.

7. SAFETY EVALUATION

Safety is evaluated as per NCI CTCAE v5.0 (see Attached Appendix III) in this study. The investigator is responsible for monitoring the safety of all subjects enrolled in this study and recording any AEs and serious adverse events (SAEs).

7.1 Adverse Events

AE is defined as any untoward medical occurrence in a subject administered a pharmaceutical product which does not necessarily have a causal relationship with this treatment. AE includes any unfavorable symptoms, signs and diseases temporally but is not necessarily associated with drug administration (worsening of a pre-existing condition during the study is also considered an AE). According to the GCP, the investigator should truthfully record all AEs in the original records and copy them into the case report form (CRF) regardless of their causal relationship with the study drug.

7.2 Serious Adverse Events

SAE is one of the following AEs occurring under any dose of the study drug or at any time during observation:

- Death: Death within 30 days after receiving the study drug, or death after 30 days due to delayed toxicity of the study drug (except death due to PD and death unrelated to the study drug);
- Serious, life-threatening toxicity requiring hospitalization or prolonged hospitalization;
- Permanent disability or dysfunction;
- Secondary tumors;
- Congenital anomaly or birth defect;
- Other important medical events.

7.3 Handling of Events

7.3.1 Adverse events

In case of mild adverse events where the patient has only tolerable symptoms, symptomatic treatment or drug discontinuation is not required. When the symptoms affect normal life and are intolerable to patients, treatment discontinuation, delayed chemotherapy, or symptomatic treatment is required.

7.3.2 Serious adverse events

In case of serious adverse events, the treatment should be immediately discontinued, and emergency treatment should be given.

7.4 Documentation of Events

AEs observed during the study should be recorded in the CRF, and those observed within 30 days after the last dose should also be recorded in the CRF if they are related to the study drug. Documentation of AEs should include event description, onset and end time, severity and frequency, whether treatment is required (if yes, the treatments given should be recorded), and whether the AE is related to the study drug as judged by the investigator.

Events should be tracked until resolution. Medical documentation of AEs should be documented in source documents (including the report of test results). If the subjects discontinue treatment due to the end of the study or discharge, the investigator should send the subjects' case summary (including treatment arrangement and instructions on whether continued follow-up is required for relevant AEs) to the physician in charge of continued treatment. The above information should also be documented in the source documents.

7.5 Reporting of Serious Adverse Events

In case of any SAEs during the trial, the study site(s) should immediately take measures to ensure the safety of the subjects and report verbally to the administrative

authorities and the sponsor in a timely manner. SAEs should be reported in writing to the Department of Safety Supervision of the National Medical Products Administration, sponsor, principal investigator, the Ethics Committee and local regulatory authorities within 24 hours of their occurrence. The investigator should record the onset time, duration, measures taken and outcome of the SAE on the report, and sign and date the report.

8. DATA COLLECTION

In order to ensure the safety of subjects throughout the study and the accuracy, completeness and reliability of the data, laboratory test reports, clinical records and patient medical records should be archived in the patients' files by the investigator as source documents for the study. The conditions of each patient should be recorded in the CRF, including disease status before, during and after treatment, toxicity, disease-related symptoms, efficacy, and dose adjustment.

9. STATISTICAL ANALYSIS

9.1 Overall Design

This study adopts a single-arm design and data are analyzed by SPSS 22.0 and summarized by descriptive statistics.

9.2 Analysis Population

The analysis population includes the full analysis set, per protocol set and safety set. The total number of enrolled subjects, number of subjects in each set, and number of excluded subjects are recorded to determine the dropout rate.

- Full analysis set (FAS): Data set obtained after excluding very few subjects who have not received the study drug or significantly violated the inclusion and exclusion criteria from the ITT set according to the principle of intention-to-treat

(ITT). Efficacy analysis is performed for all enrolled patients who have received at least one dose of the study drug in this data set.

- Per protocol set (PPS): Data set obtained after excluding all FAS subjects who violated the protocol, including subjects who completed ≥ 2 cycles of treatment, complied with the trial protocol, had good compliance, did not use prohibited medications during the trial, and completed the procedures specified in the CRF. Missing data will not be imputed. Treatment efficacy will be statistically analyzed for the FAS and PPS.
- Safety analysis set (SAS): Data set obtained by excluding very few subjects who have not received the study drug or with no safety evaluation data from the ITT set, including all patients who have received at least one dose of the study drug after enrollment and with post-dose safety records. This data set is used for safety analysis.

9.3 Main Result Analysis

9.3.1 Statistics and analysis of baseline data

The results of various parameters before treatment will be described. Categorical data will be expressed in number and percent for each category. Measured data will be expressed in number of cases, mean, standard deviation, median, maximum and minimum.

9.3.2 Safety analysis

The type and severity of AEs (adverse reactions) observed during the trial will be described, including the start and end time, severity, relationship with the study drug, measures taken and outcome. The incidence of AEs (adverse reactions) will be calculated.

9.3.3 Efficacy analysis

In this single-arm study, the efficacy endpoints will be statistically analyzed using

descriptive statistics. PFS, OS, and DOR will be described using the Kaplan-Meier estimator, and median PFS, median OS, median DOR as well as ORR, DCR and 95% confidence interval will be calculated.

9.4 End of Study

The data used for final analysis will be collected until the last patient dies, has PD, or has been enrolled for 60 months, whichever occurs first.

10. SCHEDULE OF STUDY

Start date of enrollment: March 2019; End date of enrollment: March 2021; 39 subjects are planned to be enrolled. Expected date of last follow-up: March 2026.

11. QUALITY CONTROL AND QUALITY ASSURANCE

11.1 Quality Control

11.1.1 Qualification of investigators: The qualifications of investigators participating in the clinical trial should be reviewed to ensure they have the professional background and capability to conduct the clinical trial;
11.1.2 Laboratory tests: Unified laboratory test standards and requirements should be established across the laboratories of all participating hospitals in the clinical trial;
11.1.3 Training of study personnel: The study personnel should receive training prior to the initiation of the clinical trial to ensure that they fully understand the trial protocol and the parameters specified in the protocol;
11.1.4 Clinical trial monitoring: The monitors should regularly monitor the implementation and completion of the trial. They will check the completeness of case records and the accuracy of CRFs. The investigators and relevant personnel should assist the monitors to complete their work and arrange appropriate working space for

them during their visits.

11.2 Quality Assurance

The monitors are responsible for reviewing whether the implementation of the clinical trial complies with the standard operating procedures and evaluating compliance with the Good Clinical Practice and relevant regulations. The NMPA may also audit the clinical trial during or after the trial.

12. ETHICAL PRINCIPLES

Before the trial, the protocol, proposed informed consent form, and other materials prepared for the patients must be submitted to the Independent Ethics Committee (IEC) for approval and reported to the NMPA for filing. Any amendments to the protocol, other than administrative amendments, must be approved by the IEC and reported to the NMPA for filing.

The trial can only be conducted after obtaining the subjects' signed informed consent forms. During the trial, the rights and interests of the subjects should be ensured, and the subject data should be kept confidential.

13. DATA RETENTION AND SUMMARY

The investigator must keep the original data of each patient (usually in the patient medical records), and the information in the CRF should be found in these original data, including an informed consent form signed by the patient with the trial number and title, laboratory data, and ECG results.

The study sites should retain the general information of the patients for a sufficient period of time (usually 5 years after the end of the trial) according to national regulations. General information includes 1) An Approval letter from the Ethics Committee for the protocol and all protocol amendments; 2) All original data; 3) CRF;

4) Informed consent form; 5) Any other trial-related documents.

14. MAIN REFERENCES

1. Torre LA, Bray F, Siegel RL, Ferlay J, Lortet-Tieulent J, Jemal A. Global cancer statistics, 2012. CA Cancer J Clin. 2015;65:87–108.
2. Blanchard P, Lee A, Marguet S, et al. Chemotherapy and radiotherapy in nasopharyngeal carcinoma: an update of the MAC-NPC meta-analysis. Lancet Oncol 2015; 16: 645-55.
3. Chen L1, Hu CS, Chen XZ, et al. Concurrent chemoradiotherapy plus adjuvant chemotherapy versus concurrent chemoradiotherapy alone in patients with locoregionally advanced nasopharyngeal carcinoma: a phase 3 multicentre randomised controlled trial. Lancet Oncol. 2012 Feb;13(2):163-71.
4. Melvin L K Chua, Joseph T S Wee, Edwin P Hui, et al. Nasopharyngeal carcinoma. Lancet. 2016 Mar 5;387(10022):1012-1024.
5. Tang LQ, Chen QY, Fan W, et al. Prospective study of tailoring whole-body dual-modality fluorodeoxyglucose positron emission tomography/computed tomography with plasma Epstein-Barr virus DNA for detecting distant metastasis in endemic nasopharyngeal carcinoma at initial staging. J Clin Oncol 2013; 31: 2861-69.
6. Wei WI, Sham JS. Nasopharyngeal carcinoma. Lancet 2005; 365: 2041-54.
7. Decker DA, Drelichman A, Al-Sarraf M, Crissman J, Reed ML.Chemotherapy for nasopharyngeal carcinoma: a ten-year experience. Cancer 1983; 52:602–5.
8. Chi KH, Chan WK, Cooper DL, Yen SH, Lin CZ, Chen KY. A phase II study of outpatient chemotherapy with cisplatin, 5-Fu, and leucovorin in nasopharyngeal carcinoma. Cancer 1994; 73:247–52.
9. Au E, Ang PT. A phase II trial of 5-Fu and cisplatin in recurrent or metastatic nasopharyngeal carcinoma. Ann Oncol 1994; 5:87–9.

10. Ma BB, Hui EP, Chan AT. Systemic approach to improving treatment outcome in nasopharyngeal carcinoma: current and future directions. Cancer Sci 2008; 99:1311–1318.
11. Chen C, Wang FH ,An X, et al. Triplet combination with paclitaxel, cisplatin and 5-FU is effective in metastatic and/or recurrent nasopharyngeal carcinoma. Cancer Chemother Pharmacol, 2013, 71(2):371-8.
12. Long GX, Lin JW, Liu DB, et al. Single-arm, multi-centre phase II study of lobaplatin combined with docetaxel for recurrent and metastatic nasopharyngeal carcinoma patients. Oral Oncol, 2014, 50(8):717-20.
13. Li Zhang，Yan Huang，Shao dong Hong，et al. Gemcitabine plus cisplatin versus fluorouracil plus cisplatin in recurrent or metastatic nasopharyngeal carcinoma: a multicentre，randomised，open-label，phase 3 trial[J].Lancet 2016,388:1883–1892.
14. Zhang L, Zhang Y, Huang PY, Xu F, Peng PJ, Guan ZZ. Phase II clinical study of gemcitabine in the treatment of patients with advanced nasopharyngeal carcinoma after the failure of platinum-based chemotherapy. Cancer Chemother Pharmacol. 2008;61(1):33-8.
15. Kyzas PA, Cunha IW, Ionnidis JPA. Prognostic significance of vascular endothelial growth factor immunohistochemical expression in head and neck squamous cell carcinoma: A meta-analysis. Clin Cancer Res, 2005, 11: 1434-1440.
16. Wakisaka N, Wen Q, Yoshizaki T, et al. Association of vascular endothelial growth factor expression with angiogenesis and lymph node metastasis in nasopharyngeal carcinoma. Laryngoscope, 1999,810-814.
17. Qian CN, Zhang CQ, Guo X, et al. Elevation of serum vascular endothelial growth factor in male patients with metastatic nasopharyngeal carcinoma. Cancer, 2000, 88: 255-61.
18. Krishna SM, James S, Balaram P. Expression of VEGF as prognosticator in primary nasopharyngeal cancer and its relation to EBV status. Virus Res. 2006,85-90.
19. Druzgal CH, Chen Z, Yeah NT. A pilot study of longitudinal serum cytokine and

angiogenesis factor levels as markers of therapeutic response and survival in patients with head and neck squamous cell carcinoma. Head Neck. 2005,771-784.

20. Lee NY, et al. Addition of bevacizumab to standard chemoradiation for locoregionally advanced nasopharyngeal carcinoma (RTOG 0615): a phase 2 multi-institutional trial. Lancet Oncol 2012; 13: 172-80.
21. Xue C, et al. Phase II study of sorafenib in combination with cisplatin and 5-fluorouracil to treat recurrent or metastatic nasopharyngeal carcinoma. Annals of Oncology, 2013; 24: 1055-1061.
22. Hui EP, et al. Hemorrhagic complications in a phase II study of sunitinib in patients of nasopharyngeal carcinoma who has previously received high-dose radiation. Annals of Oncology, 2011; 22: 1280-1287.
23. Yongkun Sun, Wei Niu, Feng Du, et al. Safety, pharmacokinetics, and antitumor properties of anlotinib, an oral multi-target tyrosine kinase inhibitor, in patients with advanced refractory solid tumors. J Hematol Oncol. 2016 Oct 4;9(1):105.
24. Baohui Han, Kai Li, Yizhuo Zhao, et al. Anlotinib as a third-line therapy in patients with refractory advanced non-small-cell lung cancer: a multicentre, randomised phase II trial (ALTER0302). Br J Cancer. 2018 Mar 6;118(5):654-661.
25. Baohui Han, Kai Li, Qiming Wang, et al. Efficacy and safety of third-line treatment with anlotinib in patients with refractory advanced non-small-cell lung cancer (ALTER-0303): a randomised, double-blind, placebo-controlled phase 3 study. The Lancet Oncology, Vol. 18, S3
26. Christine Elser, Lillian L. Siu, Eric Winquist, et al. Phase II Trial of Sorafenib in Patients With Recurrent or Metastatic Squamous Cell Carcinoma of the Head and Neck or Nasopharyngeal Carcinoma. J Clin Oncol. 2007 Aug 20;25(24):3766-73.
27. Ma B, Hui EP, King A, To KF, Mo F, Leung SF, et al. A phase II study of patients with metastatic or locoregionally recurrent nasopharyngeal carcinoma and evaluation of plasma Epstein-Barr virus DNA as a biomarker of efficacy.

Cancer Chemother Pharmacol. 2008;62(1):59-64.
28. Lim WT, Ng QS, Ivy P, Leong SS, Singh O, Chowbay B, et al. A Phase II study of pazopanib in Asian patients with recurrent/metastatic nasopharyngeal carcinoma. Clin Cancer Res. 2011;17(16):5481-9.

15. APPENDIX

Appendix I Staging of Nasopharyngeal Carcinoma (AJCC/UICC 8th Edition)

Primary tumor (T)

Tx Primary tumor cannot be assessed

Tis Carcinoma in situ

T0 No tumor identified, but EBV-positive cervical node(s) involvement

T1 Tumor confined to nasopharynx, or extension to oropharynx and/or nasal cavity without parapharyngeal involvement*

T2 Tumor with extension to parapharyngeal space and/or adjacent soft tissue involvement (medial pterygoid, lateral pterygoid, prevertebral muscles)

T3 Tumor with infiltration of bony structures at skull base, cervical vertebra, pterygoid structures, and/or paranasal sinuses

T4 Tumor with intracranial extension, involvement of cranial nerves, hypopharynx, orbit, parotid gland, and/or extensive soft tissue infiltration beyond lateral surface of the lateral pterygoid muscle

Regional lymph nodes (N)

Nx Regional lymph nodes cannot be assessed

N0 No regional lymph node metastasis

N1 Unilateral cervical, unilateral or bilateral retropharyngeal lymph nodes, above the caudal border of cricoid cartilage; ≤ 6 cm

N2 Bilateral metastasis in lymph node(s), 6 cm or less in greatest dimension, above the caudal border of cricoid cartilage

N3 > 6 cm and/or below caudal border of cricoid cartilage (regardless of laterality)

Distant metastasis (M)

M0 No distant metastasis

M1 Distant metastasis

Stage 0 Tis N0 M0

Stage I T1 N0 M0

Stage II T0–1 N1 M0, T2 N0–1M0

Stage III T0–2 N2 M0, T3 N0–2 M0

Stage IVA T0–3 N3 M0 or T4 N0–3 M0

Stage IVB Any T, N and M1

Appendix II Response Evaluation Criteria in Solid Tumors (RECIST 1.1)

1. Measurability of tumor at baseline

1.1 Definition

At baseline, tumor lesions/lymph nodes will be categorized measurable or non-measurable as follows:

1.1.1 Measurable lesions

Tumor lesions: Must be accurately measured in at least one dimension (the longest diameter in the plane of measurement is to be recorded) with a minimum size of:

1) 10 mm for CT scan (CT slice thickness is not more than 5 mm)

2) 10 mm for routine clinical instruments (tumor lesions that cannot be accurately measured with calipers should be recorded as unmeasurable)

3) 20 mm for chest X-ray

4) Malignant lymph nodes: Lymph nodes are pathologically enlarged and measurable, and the short axis of an individual lymph node in CT scan must be ≥ 15 mm (the recommended CT slice thickness is not more than 5 mm). At baseline and during follow-up, only the short axis will be measured and followed.

1.1.2 Non-measurable lesions

All other lesions, including small lesions (the longest axis < 10 mm or pathological lymph nodes with short axis ≥ 10 mm to < 15 mm) , and non-measurable lesions. Lesions considered truly non-measurable include: leptomeningeal disease, ascites, pleural or pericardial effusion, inflammatory breast cancer, lymphangitic involvement of skin or lung and abdominal masses identified by a physical exam that is not measurable by reproducible imaging techniques, and cystic lesions.

1.1.3 Special considerations on lesion measurement

Bone lesions, cystic lesions, and lesions previously treated with local therapy require particular comment:

Bone lesions:

- Bone scan, PET scan or plain films are not considered adequate radiological techniques to measure bone lesions. However, these techniques can be used to confirm the presence or disappearance of bone lesions.

- Lytic bone lesions or mixed lytic/blastic lesions, with identifiable soft tissue components, that can be evaluated by cross-sectional imaging techniques such as CT or MRI can be consideredmeasurable lesions if the soft tissue component meets the definition of measurability described above;

- Blastic bone lesions are non-measurable.

Cystic lesions:

- Lesions that meet the criteria for radiographically defined simple cysts should not be considered malignant lesions (neither measurable nor non-measurable) since

they are, by definition, simple cysts;

- "Cystic lesions" thought to represent cystic metastases can be consideredmeasurable lesions, if they meet the definition of measurability described above. However, if non-cystic lesions are present in the same patient, these are preferred for selection as target lesions.

Lesions previously treated with local therapy:

- Tumor lesions situated in a previously irradiated area, or in an area subjected to other loco-regional therapy, are usually not considered measurable unless there has been demonstrated progression in the lesion. Study protocols should detail the conditions under which such lesions would be considered measurable.

1.2 Specifications by methods of measurements

Measurement of lesions

All measurements should be recorded in metric notation, using calipers if clinically assessed. All baseline, evaluations should be performed as close as possible to the treatment start and never more than 28 days (4 weeks) before the beginning of the treatment.

Evaluation method

The same method of assessment and the same technique should be used to characterize each identified and reported lesion at baseline and during follow-up. Imaging-based evaluation should always be done rather than clinical examination unless the lesion(s) being followed cannot be imaged but are assessable by clinical exam.

Clinical lesions: Clinical lesions will only be considered measurable when they are superficial and 10 mm in diameter as assessed using calipers (e.g. skin nodules). For the case of skin lesions, documentation by color photography including a ruler to estimate the size of the lesion is suggested. As noted above, when lesions can be evaluated by both clinical exam and imaging, imaging evaluation should be undertaken since it is more objective and may also be reviewed at the end of the

study.

Chest X-ray: Chest CT is preferred over chest X-ray, particularly when progression is an important endpoint, since CT is more sensitive than X-ray, particularly in identifying new lesions. However, lesions on chest X-ray may be considered measurable if they are clearly defined and surrounded by aerated lungs.

CT, MRI: CT is the best currently available and reproducible method to measure lesions selected for response assessment. This guideline has defined the measurability of lesions on CT scans based on the assumption that CT slice thickness is 5 mm or less. If the CT slice thickness is greater than 5 mm, the minimum size of a measurable lesion should be twice the slice thickness. MRI is also acceptable in certain situations (e.g., for body scans).

Ultrasound: Ultrasound is not useful in the assessment of lesion size and should not be used as a method of measurement. Ultrasound examinations cannot be reproduced in their entirety for an independent review at a later date and, because they are operator-dependent, it cannot be guaranteed that the same technique and measurements will be taken from one assessment to the next. If new lesions are identified by ultrasound in the course of the trial, confirmation by CT or MRI is advised. If there is concern about radiation exposure at CT, MRI may be used instead of CT in selected instances.

Endoscopy, laparoscopy: The utilization of these techniques for objective tumor evaluation is not advised. However, they can be useful to confirm complete pathological response when biopsies are obtained or to determine relapse in trials where recurrence following complete response or surgical resection is an endpoint.

Tumor markers: Tumor markers alone cannot be used to assess objective tumor response. If markers are initially above the upper normal limit, however, they must normalize for a patient to be considered in complete response. Because tumor markers are disease-specific, instructions for their measurement should be incorporated into protocols on a disease-specific basis. Specific guidelines for both CA-125 response (in recurrent ovarian cancer) and PSA response (in recurrent prostate cancer) have been published. In addition, the Gynecologic Cancer Intergroup has developed

CA-125 progression criteria which are to be integrated with objective tumor assessment for use in first-line trials in ovarian cancer.

Cytology/histology: These techniques can be used to differentiate between PR and CR in rare cases if required by protocol (for example, residual lesions in tumor types such as germ cell tumors, where known residual benign tumors can remain). When effusions are known to be a potential adverse effect of treatment (e.g., with certain taxane compounds or angiogenesis inhibitors), the cytological confirmation of the neoplastic origin of any effusion that appears or worsens during treatment can be considered if the measurable tumor has met criteria for response or stable disease in order to differentiate between response (or stable disease) and progressive disease.

2. Tumor response evaluation

2.1 Evaluation of target lesions

Complete response (CR): Disappearance of all target lesions. Any pathological lymph nodes (whether target or non-target) must have a reduction in the short axis to < 10 mm.

Partial response (PR): At least a 30% decrease in the sum of diameters of target lesions, taking as reference the baseline sum diameters.

Progressive disease (PD): At least a 20% increase in the sum of diameters of target lesions, taking as reference the smallest sum in the study (this includes the baseline sum if that is the smallest in the study). In addition to the relative increase of 20%, the sum must also demonstrate an absolute increase of at least 5 mm. (Note: The appearance of one or more new lesions is also considered progression).

Stable disease (SD): Neither sufficient shrinkage to qualify for PR nor sufficient increase to qualify for PD, taking as reference the smallest sum diameters while on the study.

2.2 Special notes on the assessment of target lesions

Lymph nodes: Lymph nodes identified as target lesions should always have the actual short axis measurement recorded (measured in the same anatomical plane as the

baseline examination), even if the nodes regress to below 10 mm on the study. This means that when lymph nodes are included as target lesions, the "sum" of lesions may not be zero even if complete response criteria are met, since a normal lymph node is defined as having a short axis of < 10 mm. Case report forms (CRFs) or other data collection methods may therefore be designed to have target nodal lesions recorded in a separate section where, in order to qualify for CR, each node must achieve a short axis < 10 mm. For PR, SD and PD, the actual short axis measurement of the nodes is to be included in the sum of target lesions.

Target lesions that become 'too small to measure': While on study, all lesions (nodal and non-nodal) recorded at baseline should have their actual measurements recorded at each subsequent evaluation, even when very small (e.g., 2 mm). However, sometimes lesions or lymph nodes that are recorded as target lesions at baseline become so faint on CT scan that the radiologist may not feel comfortable assigning an exact measure and may report them as being 'too small to measure'. When this occurs it is important that a value be recorded on the CRF. If it is the opinion of the radiologist that the lesion has likely disappeared, the measurement should be recorded as 0 mm. If the lesion is believed to be present and is faintly seen but too small to measure, a default value of 5 mm should be assigned. (Note: It is less likely that this rule will be used for lymph nodes since they usually have a definable size when normal and are frequently surrounded by fat such as in the retroperitoneum; however, if a lymph node is believed to be present and is faintly seen but too small to measure, a default value of 5 mm should be assigned in this circumstance as well). This default value is derived from the 5 mm CT slice thickness (but should not be changed with varying CT slice thickness). The measurement of these lesions is potentially non-reproducible, therefore providing this default value will prevent false responses or progressions based upon measurement error. To reiterate, however, if the radiologist is able to provide an actual measure, that should be recorded, even if it is below 5 mm.

Lesions that split or coalesce on treatment: When non-nodal lesions "fragment", the longest diameters of the fragmented portions should be added together to calculate the

target lesion sum. Similarly, as lesions coalesce, a plane between them may be maintained that would aid in obtaining maximal diameter measurements of each individual lesion. If the lesions have truly coalesced such that they are no longer separable, the vector of the longest diameter in this instance should be the maximal longest diameter for the "coalesced lesion".

2.3 Evaluation of non-target lesions

This section provides the definitions of the criteria used to determine the tumor response for the group of non-target lesions. While some non-target lesions may actually be measurable, they need not be measured and instead should be assessed only qualitatively at the time points specified in the protocol.

CR: Disappearance of all non-target lesions and normalization of tumor marker level. All lymph nodes must be non-pathological in size (< 10 mm short axis).

Non-CR/Non-PD: Persistence of one or more non-target lesion(s) and/or maintenance of tumor marker level above the normal limits.

PD: Unequivocal progression of existing non-target lesions. Note: The appearance of one or more new lesions is also considered progression.

2.4 Special considerations on progression assessment of non-target lesions

The concept of progression of non-target disease requires additional explanation as follows. When the patient also has measurable disease: In this setting, to achieve 'unequivocal progression' on the basis of the non-target disease, there must be an overall level of substantial worsening in non-target disease such that, even in presence of SD or PR in target disease, the overall tumor burden has increased sufficiently to merit discontinuation of therapy. A modest 'increase' in the size of one or more non-target lesions is usually not sufficient to qualify for unequivocal progression status. The designation of overall progression solely on the basis of change in non-target disease in the face of SD or PR of target disease will therefore be extremely rare.

When the patient has only non-measurable disease: This circumstance arises in some phase III trials when it is not a criterion of study entry to have measurable disease. The same general concepts apply here as noted above, however, in this instance, there

is no measurable disease assessment to factor into the interpretation of an increase in non-measurable disease burden. Because worsening in non-target disease cannot be easily quantified (by definition: if all lesions are truly non-measurable) a useful test that can be applied when assessing patients for unequivocal progression is to consider if the increase in overall disease burden based on the change in non-measurable disease is comparable in magnitude to the increase that would be required to declare PD for measurable disease: i.e. an increase in tumor burden representing an additional 73% increase in 'volume' (which is equivalent to a 20% increase diameter in a measurable lesion). Examples include an increase in a pleural effusion from 'trace' to 'large', an increase in lymphangitic disease from localized to widespread, or in some cases are described in protocols as 'sufficient to require a change in therapy'. If 'unequivocal progression' is seen, the subject should be considered to have had overall PD at that point. While it would be ideal to have objective criteria to apply to non-measurable disease, the very nature of that disease makes it impossible to do so, therefore the increase must be substantial.

2.5 New lesions

The appearance of new malignant lesions denotes disease progression; therefore, some comments on the detection of new lesions are important. There are no specific criteria for the identification of new radiographic lesions; however, the finding of a new lesion should be unequivocal: i.e. not attributable to differences in scanning technique, change in imaging modality or findings thought to represent something other than tumor (for example, some "new" bone lesions may be simply healing or flare of pre-existing lesions). This is particularly important when the patient's baseline lesions show partial or complete response. For example, necrosis of a liver lesion may be reported on a CT scan report as a "new" cystic lesion, which is not.

A lesion identified on a follow-up study in an anatomical location that was not scanned at baseline is considered a new lesion and will indicate disease progression. An example of this is the patient who has visceral disease at baseline and while on study has a CT or MRI brain ordered which reveals metastases. The patient's brain metastases are considered to be evidence of PD even if he/she did not have brain

imaging at baseline.

If a new lesion is equivocal, for example, because of its small size, continued therapy and follow-up evaluation will clarify if it represents a truly new disease. If repeat scans confirm there is definitely a new lesion, then progression should be declared using the date of the initial scan.

While FDG-PET response assessments need additional study, it is sometimes reasonable to incorporate the use of FDG-PET scanning to complement CT scanning in assessment of progression (particularly possible "new" disease). New lesions on the basis of FDG-PET imaging can be identified according to the following algorithm:

Negative FDG-PET at baseline, with a positive FDG-PET at follow-up, is a sign of PD based on a new lesion.

No FDG-PET at baseline and a positive FDG-PET at follow-up:

If the positive FDG-PET at follow-up corresponds to a new site of disease confirmed by CT, this is PD.

If the positive FDG-PET at follow-up is not confirmed as a new site of disease on CT, additional follow-up CT scans are needed to determine if there is true progression occurring at that site (if so, the date of PD will be the date of the initial abnormal FDG-PET scan).

If the positive FDG-PET at follow-up corresponds to a pre-existing site of disease on CT that is not progressing on the basis of the anatomic images, this is not PD.

2.6 Missing assessments and inevaluable designation

When no imaging/measurement is done at all at a particular time point, the patient is not evaluable (NE) at that time point. If only a subset of lesion measurements are made at an assessment, usually the case is also considered NE at that time point, unless a convincing argument can be made that the contribution of the individual missing lesion(s) would not change the assigned time point response.

2.7 Special notes on response assessment

When nodal disease is included in the sum of target lesions and the nodes decrease to 'normal' size (< 10 mm), they may still have a measurement reported on scans. This measurement should be recorded even though the nodes are normal in order not to

overstate progression should it be based on increase in size of the nodes. As noted earlier, this means that subjects with CR may not have a total sum of 'zero' on the CRF.

In trials where confirmation of response is required, repeated 'NE' time point assessments may complicate best response determination. The analysis plan for the trial must address how missing data/assessments will be addressed in determination of response and progression. For example, in most trials, it is reasonable to consider a subject with time point responses of PR-NE-PR as a confirmed response.

Subjects with a global deterioration of health status requiring discontinuation of treatment without objective evidence of disease progression at that time should be reported as 'symptomatic deterioration'. Every effort should be made to document objective progression even after discontinuation of treatment. Symptomatic deterioration is not a descriptor of an objective response: It is a reason for stopping study therapy. The objective response status of such subjects is to be determined by evaluation of target and non-target diseases as shown in Tables 1–3 below.

Conditions that define 'early progression, early death and inevaluability' are study specific and should be clearly described in each protocol (depending on treatment duration, treatment periodicity).

In some circumstances, it may be difficult to distinguish the residual disease from normal tissue. When the evaluation of complete response depends upon this determination, it is recommended that the residual lesion be investigated (fine needle aspirate/biopsy) before assigning a status of complete response. FDG-PET may be used to upgrade response to a CR in a manner similar to a biopsy in cases where a residual radiographic abnormality is thought to represent fibrosis or scarring. The use of FDG-PET in this circumstance should be prospectively described in the protocol and supported by disease-specific medical literature for the indication. However, it must be acknowledged that both approaches may lead to false positive CR due to limitations of FDG-PET and biopsy resolution/sensitivity.

Table 1. Time point response: subjects with target (+/– non-target) disease

Non-target Lesions	New Lesions	Overall Response
CR	None	CR
Non-CR or non-PD	None	Non-CR or non-PD
Not all evaluated	None	Not evaluated
Equivocal PD	Yes or No	PD
Any	Yes	PD

Note: CR=complete response, PR=partial response, SD=stable disease, PD=progressive disease, NE=inevaluable.

Table 2. Time point response: subjects with non-target disease only

Overall Response (first time point)	Overall Response (subsequent time point)	Best Overall Response
CR	CR	CR
CR	PR	SD, PD, or PR[a]
CR	SD	SD provided minimum criteria for SD duration met, otherwise, PD
CR	PD	SD provided minimum criteria for SD duration met, otherwise, PD
CR	NE	SD provided minimum criteria for SD duration met, otherwise NE
PR	CR	PR
PR	PR	PR
PR	SD	SD
PR	PD	SD provided minimum criteria for SD duration met, otherwise, PD
PR	NE	SD provided minimum criteria for SD duration met, otherwise NE
NE	NE	NE

Note: CR=complete response, PR=partial response, SD=stable disease, PD=progressive disease, NE=inevaluable. Superscript "a": If a CR is truly met at first time point, then any disease seen at a subsequent time point, even disease meeting PR criteria relative to baseline, makes the disease PD at that point (since disease must have reappeared after CR). Best response would depend on whether minimum duration for SD was met. However, sometimes 'CR' may be claimed when subsequent scans suggest small lesions were likely still present and in fact, the patient had PR, not CR at the first time point. Under these circumstances, the original CR should be changed to PR and the best response is PR.

Table 3. Best overall response when confirmation of CR and PR required

Target Lesions	Non-target Lesions	New Lesions	Overall Response
CR	CR	None	CR
CR	Non-CR/Non-PD	None	PR
CR	Not evaluated	None	PR
PR	Non-PD or not all evaluated	None	PR
SD	Non-PD or not all evaluated	None	SD
Not all evaluated	Non-PD	None	NE
Target lesions	Non-target lesions	New lesions	Overall response
CR	CR	None	CR
CR	Non-CR/Non-PD	None	PR
CR	Not evaluated	None	PR
PR	Non-PD or not all evaluated	None	PR
SD	Non-PD or not all evaluated	None	SD
Not all evaluated	Non-PD	None	NE

Note: "Non-CR/non-PD" is preferred over "SD" for non-target disease. Since SD is increasingly used as an endpoint for assessment of efficacy, non-CR/non-PD response

is developed for situations where there are no criteria for the absence of measurable lesions.

For equivocal findings of progression (e.g., very small and uncertain new lesions; cystic changes or necrosis in existing lesions), treatment may continue until the next scheduled assessment. If at the next scheduled assessment, progression is confirmed, the date of progression should be the earlier date when progression was suspected

2.8 Confirmatory measurement/duration of response

Confirmation

In non-randomized trials where response is the primary endpoint, confirmation of PR and CR is required to ensure responses identified are not the result of measurement error. In randomized trials or studies where stable disease or progression is the primary endpoints, confirmation of response is not required since it will not add value to the interpretation of trial results. In the case of SD, measurements must have met the SD criteria at least once after trial entry at a minimum interval (in general not less than 6 to 8 weeks) that is defined in the trial protocol.

Duration of overall response

The duration of overall response is measured from the time measurement criteria are first met for CR/PR (whichever is first recorded) until the first date that recurrent or progressive disease is objectively documented (taking as reference for progressive disease the smallest measurements recorded on study). The duration of overall complete response is measured from the time measurement criteria are first met for CR until the first date that recurrent disease is objectively documented.

Duration of stable disease

Stable disease is measured from the start of the treatment (in randomized trials, from date of randomization) until the criteria for progression are met, taking as reference the smallest sum on study (if the baseline sum is the smallest, this is the reference for calculation of PD). The clinical relevance of the duration of stable disease varies in

different studies and diseases. If the proportion of patients achieving stable disease for a minimum period of time is an endpoint of importance in a particular trial, the protocol should specify the minimum time interval required between two measurements for determination of stable disease.

Note: The duration of response and stable disease as well as the progression-free survival (PFS) are influenced by the frequency of follow-up after baseline evaluation. It is not in the scope of this guideline to define a standard follow-up frequency. The frequency should take into account many parameters including disease types and stages, treatment periodicity, and standard practice. However, these limitations of the precision of the measured endpoint should be taken into account if comparisons between trials are to be made.

2.9 PFS/TTP

Phase III trials in advanced cancers are increasingly designed to evaluate PFS or time to progression (TTP) as the primary outcome of interest. Assessment of progression is relatively straightforward if the protocol requires all subjects to have measurable disease. Increasingly, trials allow entry of both subjects with measurable disease as well as those with non-measurable disease only. In this circumstance, care must be taken to explicitly describe the findings which would qualify for PD for those subjects without measurable lesions. Because the date of progression is subject to ascertainment bias, the timing of investigations in study arms should be the same.

Appendix III NCI-CTCAE 5.0 Evaluation Criteria for Toxic Side Effects of Tumor Drugs

Immune system disorders						
Adverse Event	Abbreviation	1	2	3	4	5
Allergic reaction/hypersensitivity (including drug fever)	Allergic reaction	Transient flushing or drug eruption; drug fever < 38°C (< 100.4°F)	Rash; flushing; drug eruption; dyspnea; drug fever ≥ 38°C (≥ 100.4°F)	Symptomatic bronchospasm, with or without drug eruption; intravenous administration indicated; allergy-related edema/angioedema; hypotension	Allergic shock	Death
Note: Drug eruptions shown to be allergic reaction or hypersensitivity are graded as allergic reaction/hypersensitivity (including drug fever). Other considerations: Cytokine release syndrome/acute infusion reaction						
Blood/bone marrow disorders						
Hemoglobin	Hemoglobin	<LLN-6.2mmol/L<br><LLN-100g/L	<6.2-4.9 mmol/L<br><100-80 g/L	<4.9-4.0 mmol/L<br><80-65 g/L	<4.0 mmol/L<br><65 g/L	Death
White blood cells (all)	White blood cells	<LLN-3000/mm^3<br><LLN-3.0×10^9/L	<3000-2000/mm^3<br><3.0-2.0×10^9/L	<2000-1000/mm^3<br><2.0-1.0×10^9/L	<1000/mm^3<br><1.0×10^9/L	Death
Lymphopenia	Leukopoenia	<LLN-800/mm^3<br><LLN-0.8×10^9/L	<800-500/mm^3<br><0.8-0.5×10^9/L	<500-200/mm^3<br><0.5-0.2×10^9/L	<200/mm^3<br><0.2×10^9/L	Death
Neutrophils/granulocytes (ANC/AGC)	Neutrophil granulocytes	<LLN-1500/mm^3<br><LLN-1.5×10^9/L	<1500-1000/mm^3<br><1.5-1.0×10^9/L	<1000-500/mm^3<br><1.0-0.5×10^9/L	<500/mm^3<br><0.5×10^9/L	Death
Platelet	Platelet	<LLN-75000/mm^3<br><LLN-75.0×10^9/L	<75000-50000/mm^3<br><75.0-50.0×10^9/L	<50000-25000/mm^3<br><50.0-25.0×10^9/L	<25000/mm^3<br><25.0×10^9/L	Death
Cardiac arrhythmia						
Supraventricular and nodal arrhythmias<br>- Options:<br>- Atrial fibrillation<br>- Atrial flutter<br>- Atrial tachycardia/paroxysmal atrial tachycardia<br>- Nodal/junctional<br>- Sinus arrhythmia<br>- Sinus bradycardia<br>- Sinus tachycardia	Supraventricular arrhythmia<br>- Supraventricular premature beats (premature atrial contraction; premature contraction of atrioventricular node/atrioventricular junction area)<br>- Supraventricular tachycardia	Asymptomatic, intervention not indicated	Non-urgent intervention indicated	Symptomatic and incompletely controlled medically, or controlled with device (e.g., pacemaker)	Life threatening (such as arrhythmia, hypotension, syncope, shock related to CHF)	Death

- Supraventricular arrhythmia NOS						
Overview of adverse cardiac events						
Left ventricular systolic dysfunction	Left ventricular systolic dysfunction	Asymptomatic, ejection fraction (EF) < 60%–50% and systolic fraction (SF) < 30%–24% at rest	Asymptomatic, EF < 50%–40% and SF < 24%–15% at rest	Symptomatic chronic heart failure, responsive to intervention, EF < 40%–20%; SF < 15%	Refractory chronic heart failure or poorly controlled, EF < 20%; intervention indicated, such as left ventricular assist device, left ventricular partial resection or heart transplant	Death
Systemic symptoms						
Tiredness (weakness, somnolence, listless)	Tiredness	Mild tiredness compared to baseline	Moderate tiredness, or causing difficulty in engaging in some activities of daily living (ADL)	Severe tiredness, limiting ADL	Causing disability	—
Fever (no neutropenia, which is defined as ANC < 1.0 × 10^9/L	Pyrexia	38.0–39.0°C (100.4-102.2°F)	>39.0–40.0°C (102.3－104.0°F)	> 40.0°C (> 104.0°F) for ≤ 24 h	> 40.0°C (> 104.0°F) for > 24 h	
Weight loss	Weight loss	5% to < 10% decrease from baseline; intervention not indicated	10% to < 20% decrease from baseline; nutritional support indicated	≥ 20% decrease from baseline; tube feeding or total parenteral nutrition (TPN) indicated	—	—
Dermatosis/skin disorders						
Alopecia/atrichia (scalp or body)	Alopecia	Sparse or alopecia areata	Complete (bald)	—	—	—
Injection site reaction/extravasation change Other considerations: Allergic reaction/hypersensitivity (including drug fever); ulcer	Injection site reaction	Pain; pruritus; erythema	Pain or swelling with inflammation or phlebitis	Severe ulcer or necrosis; surgical treatment indicated	—	—
Pruritus/itchy Other considerations: Rash/peeling	Pruritus	Mild or localized	Intense or extensive	Intense or extensive, limiting ADL	—	—
Rash/peeling	Rash	Macule or papule or	Macule or papule or	Severe systemic erythroderma	Systemic exfoliative,	Death

Note: Rash/peeling may be used for GVHD		erythema, without associated symptoms	erythema, with pruritus or other associated symptoms; localized peeling or other lesions, < 50% body surface area (BSA)	or macula, papule or blister; peeling range ≥ 50% BSA	ulcerative or bullous dermatitis	
Gastrointestinal disorders						
Appetite loss<br>Other considerations: Weight loss	Appetite loss	Appetite loss and unchanged eating habits	Oral intake changed, with no significant weight loss or malnutrition; oral nutritional supplementation indicated	Causing significant weight loss or malnutrition (e.g., inadequate oral intake of calories and/or fluids); intravenous (IV) infusion, tube feeding, or TPN indicated	Life threatening consequences	Death
Colitis<br>Other considerations: Bleeding, GI-Option	Colitis	Asymptomatic, pathological or radiological changes only	Abdominal pain, blood or mucus in stool	Abdominal pain, fever, change in bowel habits with ileus; peritoneal signs	Life threatening consequences (such as perforation, bleeding, ischaemia, necrosis, toxic megacolon)	Death
Diarrhea<br>Note: Diarrhea includes those of small bowel or colonic origin, and/or ostomy diarrhea<br>Other considerations: Dehydration; hypotension	Diarrhea	An increase in the number of bowel movements per day of < 4 times and a mild increase in ostomy output from baseline	An increase in the number of bowel movements per day of 4–6 times from baseline; IV infusion < 24 h; moderate increase in ostomy output from baseline; not limiting ADL	An increase in the number of bowel movements per day of ≥ 7 times ; fecal incontinence; IV infusion ≥ 24 h; hospitalization; severe increase in ostomy output from baseline; limiting ADL	Life-threatening consequences (e.g., hemodynamic compromise)	Death
Mucositis/stomatitis (clinical examination)<br>- Options:<br>- Anus<br>- Esophagus<br>- Large intestine<br>- Larynx<br>- Oral cavity	Mucositis (clinical examination)<br>- Options<br>- Pharynx<br>- Rectum<br>- Small intestine<br>- Stomach<br>- Trachea	Mucosal erythema	Patchy ulceration or pseudomembrane formation	Confluent ulcer or pseudomembrane formation; minor post-traumatic hemorrhage	Tissue necrosis; massive spontaneous hemorrhage; life-threatening consequences	Death
Nausea	Nausea	Appetite loss and	Reduced oral intake	Inadequate oral intake of	Life threatening	Death

Other considerations: appetite loss; vomiting		unchanged eating habits	without significant weight loss, dehydration, or malnutrition; IV infusion for < 24 h indicated	calories or fluids; IV infusion, tube feeding, or TPN for ≥ 24 h indicated	consequences	
Vomiting<br>Other considerations: Dehydration	Vomiting	1 episode within 24 hours	2 to 5 episodes within 24 hours; IV infusion for < 24 h indicated	≥ 6 episodes within 24 hours; IV infusion or TNP for ≥ 24 h indicated	Life threatening consequences	Death
Hepatobiliary disorders						
Hepatic dysfunction/failure (clinical)	Hepatic dysfunction	—	Jaundice	Biliary tremor	Hepatic encephalopathy or coma	Death
Note: Jaundice is not an AE, but it can occur with hepatic dysfunction or bile duct obstruction and is graded as a result of hepatic dysfunction/failure or increased bilirubin. Other considerations: Bilirubin (hyperbilirubinemia)						
Nervous system disorders						
Peripheral neuropathy (disease involving the peripheral nerves (sixth cranial nerve))		Asymptomatic, clinical or diagnostic observations only, intervention not indicated	Moderate symptoms: limiting instrumental ADL	Severe symptoms: limiting self-care ADL	—	—
Infections						
Febrile neutropenia (fever of unknown origin without clinically or microbiologically documented infection) (ANC < 1.0×10^9/L, fever ≥ 38.5°C)	Febrile neutropenia	—	—	Existence of symptoms	Life-threatening consequences (e.g., septic shock, hypotension, acidosis, necrosis)	Death
Other considerations: Neutrophils/granulocytes (ANC/AGC)						
Infections (clinically or microbiologically documented) with Grade 3 or 4 neutrophils (ANC < 1.0×10^9/L)	Infections (clinically documented)	—	Localized; local intervention indicated	IV antibiotic, antifungal, or antiviral intervention indicated; radiologic or operative intervention indicated	Life-threatening consequences (e.g., septic shock, hypotension, acidosis, necrosis)	Death
Note: Fever without documented infection with grade 3 or 4 neutrophils is graded as febrile neutropenia (fever of unknown origin without clinically or microbiologically documented infection) Other considerations: Neutrophils/granulocytes (ANC/AGC)						
Metabolism disorders						
Acidosis (metabolic or respiratory)	Acidosis	7.3 ≤ PH < normal value	—	pH<7.3	PH < 7.3 with life-threatening	Death

					consequences	
Albumin, low serum level (hypoalbuminaemia)	Hypoalbuminaemia	<LLN-3g/dL<br><LLN-30g/L	<3-2g/dL<br><30-20g/L	<2g/dL<br><20g/L	—	Death
ALT, SGPT (serum glutamic-pyruvic transaminase)	ALT	>ULN-2.5×ULN	>2.5-5.0×ULN	>5.0-20.0×ULN	>20.0×ULN	—
AST, SGOT (serum glutamic oxaloacetic transaminase)	AST	>ULN-2.5×ULN	>2.5-5.0×ULN	>5.0-20.0×ULN	>20.0×ULN	—
Bilirubin (hyperbilirubinemia)		>ULN-1.5×ULN	>1.5-3.0×ULN	>3.0-10.0×ULN	>10.0×ULN	—
Creatinine	Creatinine	>ULN-1.5×ULN	>1.5-3.0×ULN	>3.0-6.0×ULN	>6.0×ULN	
Note: For pediatric patients, adjust according to age-appropriate levels Other considerations: Glomerular filtration rate						
GGT (gamma-glutamyl transpeptidase)	GGT	>ULN-2.5×ULN	>2.5-5.0×ULN	>5.0-20.0×ULN	>20.0×ULN	—
Proteinuria	Proteinuria	1 + or 0.15–1.0 g/24 h	2 + to 3 + or > 1.0–3.5 g/24 h	4 + or 3.5 g/24 h	Nephrotic syndrome	Death
Pain						
Pain	Pain	Mild pain, not affecting function	Moderate pain: Pain or pain medication affects function but not ADL	Severe pain: Pain or pain medication severely affects ADL	Causing disability	—
Lung/upper respiratory tract disorders						
Pulmonary fibrosis (radiographic changes)	Pulmonary fibrosis	Minor radiographic changes (or patchy or bilateral basal lung changes), with < 25% of the entire lung volume estimated to be fibrotic based on radiographic findings.	Patchy or bilateral basal lung changes, with 25% to < 50% of the entire lung volume estimated to be fibrotic based on radiographic findings.	Intensive or extensive infiltration/consolidation, with 50% to < 75% of the entire lung volume estimated to be fibrotic based on radiographic findings.	≥ 75% of the entire lung volume is estimated to be fibrotic based on radiographic findings; honeycomb changes	Death
Note: Pulmonary fibrosis is usually a "late effect" found after 3 months of radiotherapy or multimodal combination therapy (including surgery). Pulmonary fibrosis is considered to be scarring/fibrotic lung tissue and is difficult to differentiate from pneumonia, which is usually seen within 3 months of radiotherapy or multimodal combination therapy.<br>Other considerations: Adult respiratory distress syndrome (ARDS); cough; dyspnea (air hunger); hypoxia; infection (clinically or microbiologically documented) with grade 3 or 4 neutrophils (ANC < 1.0 × 10^9/L) - Option: Infection with normal ANC or Grade 1 or 2 neutrophils - Option: Infection with unspecified ANC-Option						
Syndrome						
Influenza-like syndrome	Influenza-like syndrome	Symptomatic but not affecting function	Moderate or causing difficulty for patients to engage in some ADL	Severe symptoms, limiting ADL	Causing disability	Death

Note: Influenza-like syndrome represents a collection of symptoms, including cough with catarrhal symptoms, fever, headache, listless, muscular soreness, and frailty, and is used when a collection of symptoms occurs with a single pathophysiological change.						
Vascular disorders						
Phlebitis (including superficial thrombosis)	Phlebitis	—	Existence of symptoms	—	—	—
Other considerations: Injection site reaction/altered extravasation						

Ear and labyrinth disorders					
Adverse Event	Grade 1	Grade 2	Grade 3	Grade 4	Grade 5
Hearing impaired (damage to ear structures, causing partial or complete loss of sound perception or comprehension)	Adults enrolled on a monitoring program (a 1, 2, 3, 4, 6, and 8 kHz audiogram): Threshold shift of 15–25 dB averaged at 2 continuous test frequencies in at least one ear or subjective change in the absence of a Grade 1 threshold shift.<br><br>Pediatrics (a 1, 2, 3, 4, 6 and 8 kHz audiogram): >20 dB at any frequency tested or does not meet criteria for > Grade 2	Adults enrolled on a monitoring program (a 1, 2, 3, 4, 6, and 8 kHz audiogram): Threshold shift of > 25 dB averaged at 2 continuous test frequencies in at least one ear.<br><br>Adults not enrolled in monitoring program: Hearing loss with hearing aid or intervention not indicated; limiting instrumental ADL<br><br>Pediatrics (a 1, 2, 3, 4, 6 and 8 kHz audiogram): > 20 dB at > 4 kHz	Adults enrolled on a monitoring program (a 1, 2, 3, 4, 6, and 8 kHz audiogram): Threshold shift of > 25 dB averaged at 3 continuous test frequencies in at least one ear; intervention indicated<br><br>Adults not enrolled in monitoring program: Hearing loss with hearing aid or intervention indicated; limiting self-care ADL<br><br>Pediatrics (a 1, 2, 3, 4, 6 and 8 kHz audiogram): Hearing loss sufficient to indicate therapeutic intervention, including hearing aids; > 20 dB at 3 kHz and above in one ear; additional speech-language related services indicated	Adults: Profound bilateral hearing loss (> 80 dB at 2 kHz and above); non-serviceable hearing<br><br>Pediatrics: Audiologic indication for cochlear implant and additional speech-language related services indicated	—
Middle ear inflammation (inflammation and redness of the middle ear)	Serous otitis	Serous otitis: Medical intervention indicated	Mastoiditis: Necrosis of canal soft tissue or bone	Life-threatening: Urgent intervention indicated	Death
Tinnitus (disorder characterized by noise in ears, such as ringing, buzzing, roaring, or cracking)	Mild symptoms; intervention not indicated	Moderate symptoms: Limiting instrumental ADL	Severe symptoms: Limiting self-care ADL	—	—
Vertigo (a sensation as if the external world were revolving around the patient or as if he himself were revolving in space)	Mild symptoms	Moderate symptoms: Limiting instrumental ADL	Severe symptoms: Limiting self-care ADL	—	—

Appendix IV ECOG Scoring Criteria for Patient Quality of Life Evaluation

0	Normal activities
1	Mild symptoms, capable of all self-care and restricted in physically strenuous activity
2	Tolerable tumor symptoms, capable of all self-care, but < 50% in bed during the day
3	Severe tumor symptoms, > 50% in bed during the day but not bedbound, and capable of only limited self-care
4	Bed bound
5	Death

Appendix V FACT-H&N (V4) Quality of Life Questionnaire

The following reflect the conditions of patients with diseases similar to yours. Think back to your past **7** days and tick the number that best matches your condition. There is no right or wrong answer, please answer all questions intuitively

Physical well-being (**PWB**) in the past 7 days:

GP1 I had low energy

0. Not at all 1. A little bit 2. Somewhat 3. Quite a bit 4. Very much

GP2 I had nausea

0. Not at all 1. A little bit 2. Somewhat 3. Quite a bit 4. Very much

GP3 It was difficult for me to meet the needs of my family because of my poor health

0. Not at all 1. A little bit 2. Somewhat 3. Quite a bit 4. Very much

GP4 I had pain

0. Not at all 1. A little bit 2. Somewhat 3. Quite a bit 4. Very much

GP5 I was affected by side effects of treatment

0. Not at all 1. A little bit 2. Somewhat 3. Quite a bit 4. Very much

GP6 Generally, I felt like a patient

0. Not at all 1. A little bit 2. Somewhat 3. Quite a bit 4. Very much

GP7 I had to be in bed

0. Not at all 1. A little bit 2. Somewhat 3. Quite a bit 4. Very much

Social/Family well-being (**SWB**) in the past 7 days:

GS1 I felt close to my friends

0. Not at all 1. A little bit 2. Somewhat 3. Quite a bit 4. Very much

GS2 I got emotional support from my family

0. Not at all 1. A little bit 2. Somewhat 3. Quite a bit 4. Very much

GS3 I got support from my friends and neighbors

0. Not at all 1. A little bit 2. Somewhat 3. Quite a bit 4. Very much

GS4 My family understood my illness

0. Not at all 1. A little bit 2. Somewhat 3. Quite a bit 4. Very much

GS5 I was satisfied with family communication about my illness

0. Not at all 1. A little bit 2. Somewhat 3. Quite a bit 4. Very much

If you have a partner or have sex, please answer items 13 and 14, otherwise, you can leave it blank.

GS6 I felt close to my partner

0. Not at all 1. A little bit 2. Somewhat 3. Quite a bit 4. Very much

GS7 I was satisfied with my sex life

0. Not at all 1. A little bit 2. Somewhat 3. Quite a bit 4. Very much

Emotional well-being (**EWB**) in the past 7 days:

GE1 I felt sad

0. Not at all 1. A little bit 2. Somewhat 3. Quite a bit 4. Very much

GE2 I was satisfied with my ability to cope with the disease

0. Not at all 1. A little bit 2. Somewhat 3. Quite a bit 4. Very much

GE3 I am losing hope in the fight against my illness

0. Not at all 1. A little bit 2. Somewhat 3. Quite a bit 4. Very much

GE4 I felt nervous

0. Not at all 1. A little bit 2. Somewhat 3. Quite a bit 4. Very much

GE5 I was worried about dying

0. Not at all 1. A little bit 2. Somewhat 3. Quite a bit 4. Very much

GE6 I worried that my disease would get worse

0. Not at all 1. A little bit 2. Somewhat 3. Quite a bit 4. Very much

Functional well-being (**FWB**) in the past 7 days:

GF1 I was able to work (including housework)

0. Not at all 1. A little bit 2. Somewhat 3. Quite a bit 4. Very much

GF2 My work (including housework) was fulfilling

0. Not at all 1. A little bit 2. Somewhat 3. Quite a bit 4. Very much

GF3 I was able to enjoy life

0. Not at all 1. A little bit 2. Somewhat 3. Quite a bit 4. Very much

GF4 I had accepted my illness

0. Not at all 1. A little bit 2. Somewhat 3. Quite a bit 4. Very much

GF5 I slept well

0. Not at all 1. A little bit 2. Somewhat 3. Quite a bit 4. Very much

GF6 I was enjoying the things I usually do for fun

0. Not at all 1. A little bit 2. Somewhat 3. Quite a bit 4. Very much

GF7 I was content with my quality of life right now

0. Not at all 1. A little bit 2. Somewhat 3. Quite a bit 4. Very much

Additional questions (**FACT-HN**) in the past 7 days:

hn1 I was able to eat the foods that I like

0. Not at all 1. A little bit 2. Somewhat 3. Quite a bit 4. Very much

hn2 My mouth was dry

0. Not at all 1. A little bit 2. Somewhat 3. Quite a bit 4. Very much

hn3 I had trouble breathing

0. Not at all 1. A little bit 2. Somewhat 3. Quite a bit 4. Very much

hn4 My voice had its usual quality and strength

0. Not at all 1. A little bit 2. Somewhat 3. Quite a bit 4. Very much

hn5 I was able to eat as much food as I want

0. Not at all	1. A little bit	2. Somewhat	3. Quite a bit	4. Very much

hn6 I felt like my appearance (e.g., neck) had changed

0. Not at all	1. A little bit	2. Somewhat	3. Quite a bit	4. Very much

hn7 I could swallow naturally and easily

0. Not at all	1. A little bit	2. Somewhat	3. Quite a bit	4. Very much

hn8 I smoked

0. Not at all	1. A little bit	2. Somewhat	3. Quite a bit	4. Very much

hn9 I drank alcohol (beer, wine or liquor)

0. Not at all	1. A little bit	2. Somewhat	3. Quite a bit	4. Very much

hn10 I was able to communicate with others

0. Not at all	1. A little bit	2. Somewhat	3. Quite a bit	4. Very much

hn11 I could eat solid foods

0. Not at all	1. A little bit	2. Somewhat	3. Quite a bit	4. Very much

**FACT-H&N Area of Scoring Method**	**Number of Entries**	**Score Range**	**Scoring Method**
Physical well-being (PWB)	7	0–28	GP1+ GP2+ GP3+ GP4+ GP5+ GP6+ GP7
Social/Family well-being (SWB)	7	0–28	GS1+ GS2+ GS3+ GS4+ GS5+ GS6+ GS7
Emotional well-being (EWB)	6	0–24	GE1+ GE2+ GE3+ GE4+ GE5+ GE6
Functional well-being (FWB)	7	0–28	GF1+ GF2+ GF3+ GF4+ GF5+ GF6+ GF7
Functional Assessment of Cancer Therapy - General (FACT-G)	27	0–108	PWB+ SWB+ EWB+ FWB
Head and neck subscale (HNS)	9+2	0–36	hn1+hn2+hn3+hn4+hn5+hn6+hn7+hn10+hn11
Total score	38	0–144	PWB+ SWB+ EWB+ FWB+HNS
